# Magnetic Resonance Imaging-Based Cortical and Subcortical Volumetric Changes in Alzheimer’s Disease: Association with Clinical Severity

**DOI:** 10.3390/diagnostics16162596

**Published:** 2026-08-16

**Authors:** Aybala Neslihan Alagoz, Serap Ozturk, Sena Destan Bunul, Yonca Anık, Torehan Ozer, Guldeniz Cetin Erci

**Affiliations:** 1 Department of Neurology, Faculty of Medicine, Kocaeli University, Kocaeli 4100, Turkey; destansena@gmail.com (S.D.B.); guldenizcetinerci@gmail.com (G.C.E.); 2 Department of Neurology, Kurtalan State Hospital, Siirt 5600, Turkey; ozturkkserap@gmail.com; 3 Department of Radiology, Faculty of Medicine, Kocaeli University, Kocaeli 4100, Turkey; yoncaanik@kocaeli.edu.tr (Y.A.); torehanozer@gmail.com (T.O.)

**Keywords:** Alzheimer’s disease, mild cognitive impairment, automated MRI volumetry, hippocampal volume, cortical thickness, cerebrospinal fluid volume

## Abstract

**Background/Objectives**: This study evaluated automated magnetic resonance imaging (MRI) volumetry for characterizing structural brain changes in Alzheimer’s disease (AD), mild cognitive impairment (MCI), and cognitively healthy controls (HC), and examined its association with cognitive performance. **Methods**: This retrospective observational study included consecutively enrolled individuals aged ≥65 years. AD dementia and MCI were diagnosed according to the 2011 National Institute on Aging–Alzheimer’s Association (NIA-AA) clinical criteria. Automated volumetric analysis of structural MRI was used to obtain the total intracranial volume-normalized cortical and subcortical volumes, cerebrospinal fluid (CSF) compartments, and hemispheric asymmetry indices. Between-group comparisons were corrected using the Benjamini–Hochberg false discovery rate. Prespecified volumetric markers were evaluated using receiver operating characteristic analysis and internally validated logistic regression models. **Results**: The study included 102 participants (34 per group). Compared with HC, the AD group showed lower total cerebrum, right hippocampal, and anterior cingulate gyrus (ACgG) volumes and higher CSF volumes. Compared with MCI, the AD group exhibited lower thalamic and caudate volumes. CSF volume showed the highest individual discriminative performance for differentiating AD from HC (AUC = 0.837), whereas the combined hippocampal–ACgG–CSF model showed the best performance for differentiating MCI from HC (AUC = 0.826). After adjustment for diagnostic group, cognitive performance remained positively associated with hippocampal volume and negatively with CSF and lateral ventricular volumes. **Conclusions**: Automated MRI volumetry may support the quantitative assessment of neurodegeneration in clinically defined AD and MCI. Combined volumetric markers improved discrimination between MCI and HC, although these findings require external validation in larger, longitudinal, biomarker-characterized cohorts.

## 1. Introduction

Alzheimer’s disease (AD) is the most common cause of dementia in older adults and is a neurodegenerative disorder characterized by progressive impairment of memory, executive functions, attention, language, and activities of daily living. The disease typically presents with insidious memory complaints and gradually progresses to substantial cognitive and functional decline, compromising independent living [1]. Mild cognitive impairment (MCI) is considered an important intermediate stage between normal cognitive function and AD, during which objective biomarkers are needed to assess the disease burden and risk of clinical progression [2].

The accumulation of beta-amyloid and the formation of neurofibrillary tangles related to hyperphosphorylated tau protein are among the core biological processes underlying AD pathogenesis [3]. These pathological changes are known to emerge initially in medial temporal lobe structures, particularly the entorhinal cortex and hippocampus, and spread with disease progression to the posterior cingulate cortex, lateral temporal cortex, frontotemporoparietal neocortex, and eventually the occipital cortex [2]. This neuroanatomical pattern of propagation represents one of the key mechanisms underlying the episodic memory impairment observed in the early stages and the more widespread cognitive deficits that develop as the disease advances [4].

Structural magnetic resonance imaging (MRI) is a widely used, accessible, and noninvasive method for assessing neurodegenerative changes in AD [5]. During the disease course, atrophy becomes evident early in the medial temporal lobe structures, particularly the hippocampus and entorhinal cortex, and may subsequently extend to the temporal, parietal, and other cortical and subcortical regions as the disease progresses [2,5]. Therefore, evaluating hippocampal volume loss and local and total volume changes across different cortical and subcortical brain structures may provide a more comprehensive characterization of structural involvement in AD. However, most existing automated volumetry studies have predominantly focused on hippocampal and global brain volumes, whereas multidimensional analyses integrating cortical, subcortical, cerebrospinal fluid (CSF), and hemispheric asymmetry markers are limited [6,7].

MRI volumetry is an important imaging approach that enables the quantitative assessment of volumetric changes in brain structures. Compared with manual measurements, automated segmentation and volumetry software provide faster, standardized, and reproducible results, thereby contributing to the objective evaluation of regional brain atrophy in the diagnosis of dementia [7]. Clinical studies have shown that automated MRI volumetry may be useful in differentiating AD, MCI, and cognitively healthy controls (HC), particularly through hippocampal, cerebral, ventricular, and CSF measurements [8]. Nevertheless, volumetric MRI findings alone may not be sufficient for diagnostic decision-making; rather, they may provide more meaningful information for early-stage differentiation and prognosis when interpreted together with cognitive testing and neuropsychological assessment [9]. Furthermore, recent neuroimaging studies have suggested that cortical and subcortical atrophy across the AD spectrum does not progress in a fully symmetric manner between hemispheres and that asymmetry-based morphometric measures may provide complementary information beyond conventional volumetric analyses [10].

In this study, we aimed to compare automated MRI volumetry-derived cortical and subcortical volume measurements between HC, MCI, and AD groups across the AD spectrum and to evaluate the associations of these measurements with cognitive performance and clinical disease stage.

## 2. Materials and Methods

### 2.1. Study Design and Patient Selection

This retrospective observational study included individuals aged ≥65 years who were evaluated at the dementia outpatient clinic between 2023 and 2024, underwent brain MRI as part of routine clinical assessment, and had image quality sufficient for volumetric analysis. Participants were classified into three groups, AD, MCI, and HC, based on clinical evaluation and cognitive test results.

An a priori power analysis was performed using G*Power version 3.1.9.4. Assuming a one-way analysis of variance with three groups, an effect size of f = 0.40, a significance level of α = 0.05, and a statistical power of 1 − β = 0.95, the minimum required sample size was calculated as 102 participants (34 per group). Eligible individuals who met the predefined inclusion and exclusion criteria were included consecutively according to the order of presentation until the target sample size of 34 participants was reached for each group. No individual matching or random sampling was performed.

The diagnosis of AD dementia and MCI was established by an experienced neurologist according to the 2011 National Institute on Aging–Alzheimer’s Association (NIA-AA) clinical diagnostic criteria for AD dementia [11] and MCI due to AD [12]. Diagnoses were based on a comprehensive clinical assessment, including medical history obtained from the patient and an informant, neurological examination, assessment of cognitive complaints and objective cognitive impairment, evaluation of functional activities of daily living, and structural brain MRI. Cognitive performance was assessed using the validated Turkish version of the Mini-Mental State Examination (MMSE), reported by Güngen et al. [13].

The AD group consisted of individuals with progressive cognitive decline accompanied by significant impairment in activities of daily living, clinically compatible with AD, and an MMSE score of ≤23. The MCI group included individuals with cognitive complaints and objective cognitive impairment but preserved independence in activities of daily living, with MMSE scores between 24 and 27.

The HC group consisted of cognitively unimpaired individuals who underwent brain MRI for neurological complaints unrelated to cognitive impairment, including headache, dizziness, vertigo, tinnitus, or syncope, and who had no subjective cognitive complaints or objective cognitive impairment, preserved activities of daily living, and an MMSE score of ≥28. Selection of the control group was performed without knowledge of the automated volumetric analysis results.

The same exclusion criteria were applied uniformly across all three groups. Individuals with Parkinson’s disease, multiple sclerosis, or any neurodegenerative disorder other than AD were excluded. Additional exclusion criteria included clinically significant head trauma or major brain injury; ischemic or hemorrhagic stroke; transient ischemic attack; hypoxic–ischemic encephalopathy; encephalitis; brain abscess; intracranial mass; hydrocephalus; confluent white matter hyperintensities corresponding to Fazekas grade 3; territorial infarction; or any other structural brain abnormality that could affect regional volumetric measurements. Participants with major depressive disorder, bipolar disorder, psychotic disorder, or another major psychiatric illness; alcohol or substance dependence; or secondary metabolic or systemic causes of cognitive impairment were also excluded. MRI contraindications, including implanted devices, prostheses, metallic plates, or foreign bodies, as well as insufficient image quality for volumetric analysis, constituted additional exclusion criteria.

For all participants, age, sex, years of education, body mass index (BMI), marital status, number of children, smoking and alcohol use, comorbidities, and family history were documented. All procedures were conducted in accordance with the principles of the Declaration of Helsinki. Ethical approval was obtained from the Kocaeli University Faculty of Medicine Non-Interventional Clinical Research Ethics Committee (GOKAEK-2025/15/13; File No: 2025/366; Approval Date: 17 July 2025).

### 2.2. Structural MRI Protocol

The MRI protocol encompassed cranial MRI for all participants. Imaging was conducted using a 3 Tesla MRI scanner (Achieva, Philips Medical Systems, Best, The Netherlands) equipped with an eight-channel head-coil. The protocol included two primary axial sequences: a three-dimensional T1-weighted turbo field echo (3D T1W TFE) and a three-dimensional fluid-attenuated inversion recovery (3D FLAIR). For the T1-weighted sequence, the acquisition parameters were as follows: repetition time (TR) of 8 ms, echo time (TE) of 4 ms, slice thickness of 1 mm, no interslice gap, a field of view (FOV) of 256 mm, and a matrix size of 512 × 512. For the FLAIR sequence, the parameters included a TR of 5800 ms, a TE of 315 ms, an inversion time (TI) of 1650 ms, a slice thickness of 2 mm, no interslice gap, an FOV of 256 mm, and a matrix size of 512 × 512. The volBrain software was utilized to perform automated and standardized measurements of these brain structures. volBrain is a fully automated online brain MRI volumetry system that provides volumetric measurements of the intracranial cavity, brain tissues, subcortical structures, cerebellum, brainstem, and cerebral hemispheres. Compared with manual measurements, this automated workflow has been reported to improve standardization, reduce observer-dependent variability, and provide a reproducible framework for volumetric analysis [14]. A complete volBrain output report from a representative HC participant is provided as Supplementary Material S1.

### 2.3. Quantitative Image Analysis with volBrain

The 3D T1-weighted MRI data of all participants were retrieved from the picture archiving and communication system (PACS) and transferred to a personal computer for analysis. Images stored in the Digital Imaging and Communications in Medicine (DICOM) format were organized into separate folders for each participant. DICOM files were converted into Neuroimaging Informatics Technology Initiative (NIfTI; .nii/.nii.gz) format using the dcm2nii tool implemented in MRIcron software. The resulting NIfTI files were anonymized, appropriately compressed, and uploaded to the online VolBrain platform. Age and sex information for each participant were entered during the upload process. After processing, the platform provided automated volumetric reports in a portable document format.

All structural volume measures were normalized to the total intracranial volume (TIV) to account for interindividual differences in head size and were expressed as percentages of TIV (%TIV). Hemispheric asymmetry was quantified using the standard asymmetry index (AI) of the cortical and subcortical structures. AI was calculated as the difference between the right and left hemispheric measurements divided by their mean and expressed as a percentage:

AI (%) = 100 × (Right − Left)/[(Right + Left)/2].

This approach has been adopted by the ENIGMA Laterality Working Group in large-scale healthy population studies [15] and in the evaluation of hippocampal and amygdalar asymmetry in neurodegenerative diseases [16]. Positive AI values indicate rightward asymmetry, whereas negative values indicate leftward asymmetry.

**Statistical Analysis**: Statistical analyses were performed using IBM SPSS Statistics version 26.0 (IBM Corp., Armonk, NY, USA) and R version 4.1.2 (R Foundation for Statistical Computing, Vienna, Austria). The pROC package was used for ROC analyses and DeLong comparisons, the boot package for bootstrap resampling, the caret package for cross-validation, and the ppcor package for partial correlation analyses. The distribution of continuous variables was assessed using the Shapiro–Wilk test together with visual inspection of histograms and Q–Q plots. Demographic and cognitive continuous variables are presented as median (interquartile range [IQR]), whereas TIV-normalized volumetric measurements (%TIV) are presented as mean ± standard deviation (SD). Categorical variables are presented as number (%).

Given that most volumetric variables deviated from normality, between-group comparisons were performed using the Kruskal–Wallis test, followed by Dunn’s post hoc test with Bonferroni correction for pairwise comparisons. Effect sizes were expressed as epsilon-squared (ε^2^) and interpreted as small (≥0.01), moderate (≥0.06), or large (≥0.14). Categorical variables were compared using Pearson’s chi-square test or the Fisher–Freeman–Halton exact test when appropriate. Alcohol consumption was analysed using an exact permutation test with 10,000 permutations because of the small number of participants reporting alcohol use.

To account for multiple comparisons, the Benjamini–Hochberg false discovery rate (FDR) procedure was applied. FDR correction was performed across the 73 volumetric variables and separately across the 30 association tests. FDR-adjusted *q* values, together with the corresponding unadjusted *p* values. Demographic and clinical characteristics (Table 1) were analysed descriptively and were therefore not included in the FDR correction.

**Table 1:** Demographic and Clinical Characteristics by Cognitive Group.

Variables	AD (*n* = 34)	MCI (*n* = 34)	HC (*n* = 34)	*p*
**Demographic features**				
Age (years), median (IQR)	73.0 (67.0–79.8)	71.0 (68.0–77.0)	71.5 (68.0–74.8)	0.631
BMI (kg/m^2^), median (IQR)	24.9 (23.6–27.0)	25.4 (25.0–28.2)	25.9 (23.6–29.2)	0.391
Sex, *n* (%)				
Female	20 (58.8)	17 (50.0)	14 (41.2)	0.371
Male	14 (41.2)	17 (50.0)	20 (58.8)	
**Sociodemographic features**				
Education (years), median (IQR)	5.0 (3.5–7.8)	5.0 (5.0–8.0)	6.5 (5.0–11.0)	0.118
**Marital status, ** ***n*** ** (%)**				
Married	28 (82.4)	28 (82.4)	26 (76.5)	0.860
Single	6 (17.6)	6 (17.6)	8 (23.5)	
Children, median (IQR)	3.0 (2.0–3.8)	3.0 (2.0–3.0)	3.0 (2.0–4.0)	0.453
**Lifestyle factors**				
Smoking history (pack-years > 0), *n* (%)	14 (41.2)	15 (44.1)	16 (47.1)	0.968
Alcohol use, *n* (%)				
Never drinker	30 (88.2)	33 (97.1)	26 (76.5)	0.061
Social drinker	4 (11.8)	1 (2.9)	7 (20.6)	
Current drinker	0 (0.0)	0 (0.0)	1 (2.9)	
**Clinical features**				
Comorbidity, *n* (%)				
Present	28 (82.4)	28 (82.4)	26 (76.5)	0.860
Absent	6 (17.6)	6 (17.6)	8 (23.5)	
**Family history, ** ***n*** ** (%)**				
Present	15 (44.1) ᵃ	14 (41.2) ᵃᵇ	5 (14.7) ᵇ	0.017 *
Absent	19 (55.9)	20 (58.8)	29 (85.3)	
**Cognitive assessment**				
MMSE score, median (IQR)	18.0 (14.0–20.0) ᵃ	26.0 (25.0–26.0) ᵇ	29.0 (28.0–30.0) ᶜ	<0.001 *

AD: Alzheimer’s disease; MCI: mild cognitive impairment; HC: healthy control; BMI: body mass index; IQR: interquartile range; MMSE: Mini-Mental State Examination. Continuous variables were compared using the Kruskal–Wallis test; categorical variables using the Fisher–Freeman–Halton exact test. Alcohol use was dichotomised as any use versus non-use and tested by exact permutation test. Different superscript letters in the same row indicate significant pairwise differences (Bonferroni-adjusted Dunn test for continuous variables; pairwise Fisher exact test for categorical variables). Demographic and clinical variables are presented for descriptive purposes and were not included in the FDR correction applied to volumetric measures. * *p* < 0.05.

Associations between cognitive performance and volumetric measures were assessed using Spearman’s rank correlation coefficient. Because MMSE scores were used to support diagnostic group classification, pooled MMSE–volume correlations were supplemented by partial correlation analyses adjusted for diagnostic group and by within-group correlation analyses performed separately for the HC, MCI, and AD groups.

The discriminative performance of volumetric markers was evaluated using receiver operating characteristic (ROC) analysis. The four markers were prespecified a priori based on evidence from previous volumetric MRI studies of AD [17,18,19,20], and their selection was independent of the between-group findings in the present study. Areas under the curve (AUCs) with 95% confidence intervals, optimal cut-off values determined by the Youden index, corresponding sensitivities, and specificities were calculated for AD versus HC and MCI versus HC comparisons. To reduce overfitting, combined models were internally validated using 1000 bootstrap resamples and repeated five fold cross-validation (20 repetitions). ROC curves were compared using DeLong’s test, with the strongest individual marker serving as the reference. Given the limited sample size, multivariable models are presented as internally validated exploratory models. All statistical tests were two-sided; *p* < 0.05 was considered statistically significant, and *q <* 0.05 indicated statistical significance after FDR correction.

## 3. Results

A total of 102 patients were included with 34 in each of the AD, MCI and HC sub-groups. The clinically defined AD, MCI, and HC groups showed comparable demographic and clinical characteristics in terms of age, sex, BMI, years of education, marital status, number of children, smoking exposure, alcohol use, and comorbidity status (Table 1). A positive family history of cognitive impairment differed significantly across the groups (*p* = 0.017). Post hoc analysis showed a significant difference between the AD and HC groups (*p* = 0.046), whereas the MCI–HC comparison was not significant (*p* = 0.087). As expected, MMSE scores differed significantly among the groups (*p* < 0.001), with the lowest scores in the AD group and the highest scores in the HC group.

Total white matter volume (WMV) and normal-appearing white matter (NAWM) volume differed significantly between the groups, with higher values in the HC group and reduced volumes in the AD and MCI groups (*p* < 0.001), whereas abnormal-appearing white matter (AbAWM) volume did not differ (*p* = 0.484) (Table 2). Total and cortical gray matter (GM) volumes were comparable (*p* = 0.413 and *p* = 0.559), whereas subcortical GM volume was lowest in the AD group (*p* < 0.001). Total CSF, external CSF, and lateral ventricle (LV) volumes were highest in the AD group and lowest in the HC group (*p* < 0.05); for total and external CSF, the MCI group also differed from the HC group (*p* = 0.023 and *p* = 0.022), whereas AD–MCI contrasts were not significant. Total cerebrum volume showed the opposite pattern (*p* < 0.001), with a significant MCI–HC difference as well (*p* = 0.020). Cerebellar and brainstem volumes did not differ between the groups. All significant between-group differences remained significant after false discovery rate (FDR) correction (all *q* < 0.05), with effect sizes ranging from small to moderate (ε^2^ = 0.10–0.21).

**Table 2:** Total Brain Volumetric Measures by Cognitive Group.

Variables (%TIV)	AD (Mean ± SD)	MCI (Mean ± SD)	HC (Mean ± SD)	*p*	*q*	ε^2^
**Total** ** WM**						
Total WMV	34.29 ± 7.40 ᵃ	36.42 ± 8.10 ᵃ	40.36 ± 6.72 ᵇ	<0.001 *	0.002 †	0.163
NAWM	32.58 ± 6.93 ᵃ	35.11 ± 7.91 ᵃ	38.97 ± 6.20 ᵇ	<0.001 *	0.001 †	0.177
AbAWM	1.72 ± 1.83	1.31 ± 1.26	1.36 ± 2.10	0.484	0.681	0.000
**Total GM**						
Total GMV	42.09 ± 4.70	43.67 ± 4.43	43.59 ± 5.61	0.413	0.669	0.000
Cortical GMV	33.19 ± 4.39	33.96 ± 4.19	34.48 ± 4.83	0.559	0.681	0.000
Subcortical GMV	2.41 ± 0.49 ᵃ	3.07 ± 0.91 ᵇ	2.81 ± 0.83 ᵇ	<0.001 *	0.006 †	0.129
Cerebellar GMV	6.49 ± 1.36	6.64 ± 1.37	6.35 ± 1.27	0.545	0.681	0.000
**CSF and intracranial volume**						
Total CSF	22.22 ± 6.58 ᵃ	18.43 ± 7.30 ᵃ	14.28 ± 5.17 ᵇ	<0.001 *	<0.001 †	0.208
External CSF	17.11 ± 5.55 ᵃ	15.26 ± 5.65 ᵃ	12.00 ± 4.22 ᵇ	<0.001 *	0.004 †	0.140
LV	3.95 ± 2.39 ᵃ	2.72 ± 1.74 ᵃᵇ	2.22 ± 1.05 ᵇ	0.002 *	0.019 †	0.102
Third ventricle	0.22 ± 0.10	0.18 ± 0.09	0.17 ± 0.08	0.110	0.299	0.024
**Cerebrum and cerebellum**						
Total cerebrum	67.61 ± 6.09 ᵃ	71.11 ± 6.45 ᵃ	74.72 ± 4.25 ᵇ	<0.001 *	<0.001 †	0.209
Total cerebellum	8.17 ± 1.54	8.10 ± 1.91	8.65 ± 1.14	0.561	0.681	0.000
Brainstem	1.40 ± 0.20	1.48 ± 0.21	1.49 ± 0.18	0.064	0.203	0.035

WMV: white matter volume; NAWM: normal-appearing white matter; AbAWM: abnormal-appearing white matter; GMV: grey matter volume; LV: lateral ventricle; CSF: cerebrospinal fluid; %TIV: percentage of total intracranial volume; *q*: Benjamini–Hochberg FDR-corrected *p* value across all 73 volumetric and thickness variables; ε^2^: epsilon-squared effect size. Data are presented as mean ± SD. Groups were compared using the Kruskal–Wallis test with Bonferroni-adjusted Dunn post hoc comparisons; different superscript letters in the same row indicate significant pairwise differences. * *p* < 0.05; † *q* < 0.05.

Subcortical analyses showed significant group differences in hippocampal, thalamic, caudate, and putamen volumes (Table 3). Total and left hippocampal volumes were lower in the AD group than in the MCI and HC groups (omnibus *p* = 0.012 and *p* = 0.013, respectively), whereas no significant difference was observed between the MCI and HC groups. Right hippocampal volume also differed significantly across groups (omnibus *p* = 0.004), with lower values in the AD group than in both the MCI and HC groups. Hippocampal asymmetry did not differ between the groups (*p* = 0.665).

Total thalamic volume differed significantly across groups (omnibus *p* = 0.002), with lower values in the AD group than in both the MCI and HC groups; the AD–HC comparison was significant (*p* = 0.038). Right and left thalamic volumes also differed significantly (*p* = 0.003 for both). For the right thalamus, only the AD–MCI comparison was significant, whereas the AD–HC comparison did not reach statistical significance (*p* = 0.058). For the left thalamus, the AD group had lower volumes than both the MCI and HC groups, with a significant AD–HC difference (*p* = 0.018).

Caudate and putamen volumes differed significantly across groups (omnibus *p* = 0.006 and *p* = 0.019, respectively), with the highest values in the MCI group and no significant AD–HC differences. Amygdala, pallidum, nucleus accumbens, and ventral diencephalon (DC) volumes did not differ significantly across groups (all *p* > 0.05). Basal forebrain volume showed a significant omnibus group effect, although no pairwise comparison remained significant.

**Figure 1:**
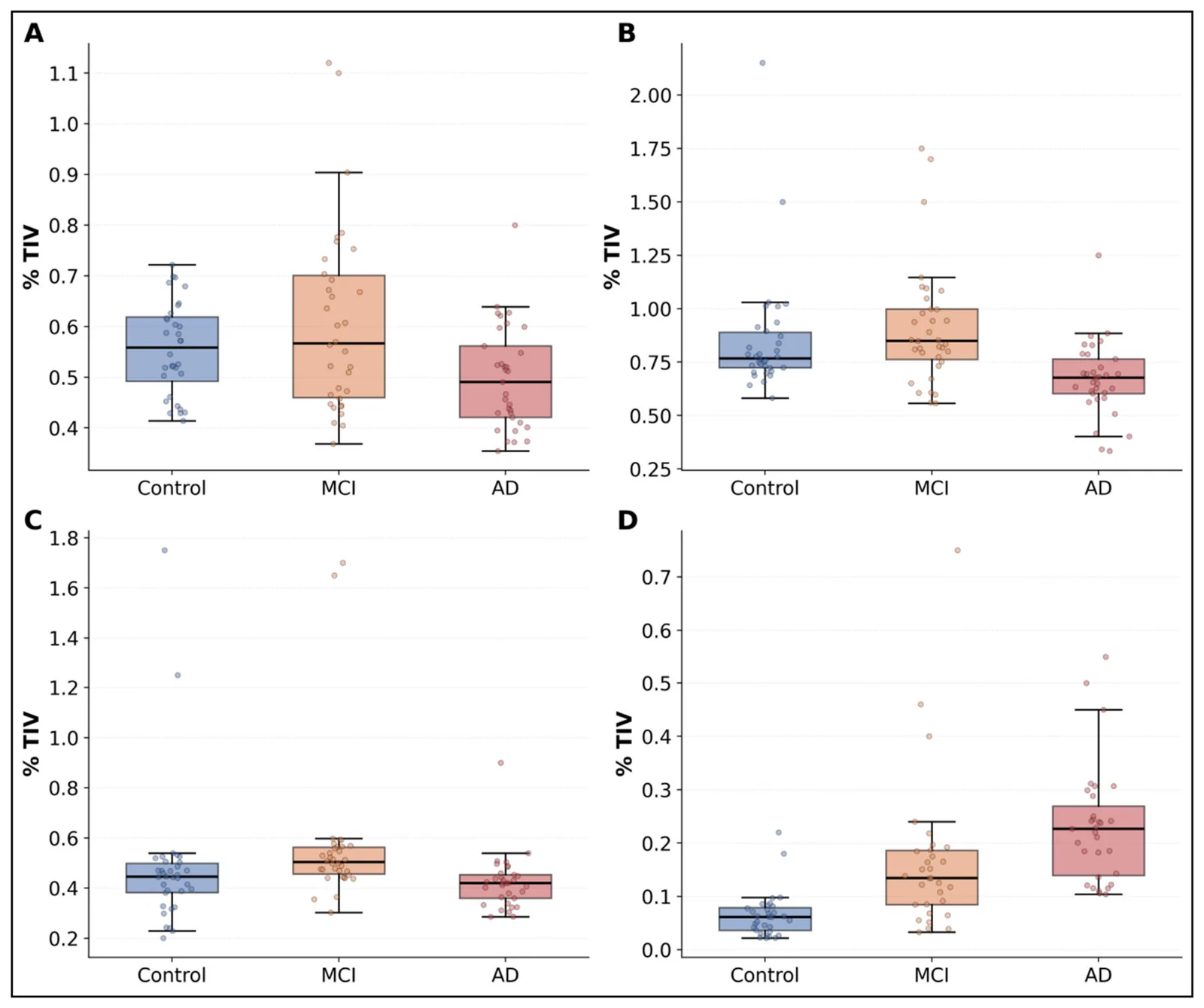
Distribution of selected subcortical and ventricular volumes (%TIV) across cognitive groups (HC, MCI, and AD/dementia): (**A**) total hippocampus, (**B**) total thalamus, (**C**) total caudate, and (**D**) lateral ventricle. Box plots show the median and interquartile range with individual data points. Group comparisons correspond to the Kruskal–Wallis and Bonferroni-adjusted Dunn post hoc results reported in Table 2 and Table 3. TIV: total intracranial volume; HC: healthy control; MCI: mild cognitive impairment; AD: Alzheimer’s disease.

After FDR correction, right hippocampal volume (*q* = 0.020), total, right, and left thalamic volumes (*q* = 0.014–0.019), and total caudate volume (*q* = 0.030) remained significant. In contrast, total and left hippocampal volumes, total putamen volume, and basal forebrain volume did not remain significant after correction (*q* = 0.052–0.145). The distributions of hippocampal, thalamic, caudate, and inferior lateral ventricular volumes are shown in Figure 1.

**Table 3:** Subcortical GM Volumes by Cognitive Group.

Variables (%TIV)	AD (Mean ± SD)	MCI (Mean ± SD)	HC (Mean ± SD)	*p*	*q*	ε^2^
**Hippocampal complex**						
Total hippocampus	0.50 ± 0.09 ᵃ	0.61 ± 0.20 ᵇ	0.57 ± 0.11 ᵇ	0.012 *	0.052	0.069
Right hippocampus	0.25 ± 0.04 ᵃ	0.31 ± 0.11 ᵇ	0.29 ± 0.06 ᵇ	0.004 *	0.020 †	0.093
Left hippocampus	0.25 ± 0.07 ᵃ	0.30 ± 0.11 ᵇ	0.28 ± 0.06 ᵇ	0.013 *	0.055	0.067
Hippocampal asymmetry (%)	1.90 ± 19.59	2.88 ± 18.01	1.25 ± 7.77	0.665	0.735	0.000
**Amygdala and limbic structures**						
Total amygdala	0.11 ± 0.04	0.12 ± 0.03	0.12 ± 0.04	0.288	0.512	0.005
Right amygdala	0.05 ± 0.02	0.06 ± 0.02	0.06 ± 0.02	0.178	0.418	0.015
Left amygdala	0.05 ± 0.02	0.06 ± 0.02	0.06 ± 0.02	0.269	0.492	0.006
Amygdalar asymmetry (%)	1.92 ± 22.15	−0.69 ± 27.21	5.67 ± 24.90	0.258	0.492	0.007
Basal forebrain	0.05 ± 0.01	0.05 ± 0.01	0.04 ± 0.02	0.042 *‡	0.145	0.044
**Thalamus**						
Total thalamus	0.66 ± 0.19 ᵃ	0.87 ± 0.31 ᵇ	0.80 ± 0.31 ᵇ	0.002 *	0.014 †	0.111
Right thalamus	0.32 ± 0.10 ᵃ	0.42 ± 0.14 ᵇ	0.39 ± 0.18 ᵃᵇ	0.003 *	0.019 †	0.098
Left thalamus	0.34 ± 0.11 ᵃ	0.45 ± 0.17 ᵇ	0.42 ± 0.14 ᵇ	0.003 *	0.019 †	0.098
Thalamic asymmetry (%)	−7.94 ± 20.11	−7.26 ± 21.91	−12.74 ± 27.28	0.557	0.681	0.000
**Striatum**						
Total putamen	0.49 ± 0.13 ᵃ	0.63 ± 0.19 ᵇ	0.54 ± 0.27 ᵃᵇ	0.019 *	0.073	0.060
Total caudate	0.42 ± 0.19 ᵃ	0.61 ± 0.36 ᵇ	0.47 ± 0.30 ᵃᵇ	0.006 *	0.030 †	0.082
Total pallidum	0.17 ± 0.04	0.16 ± 0.07	0.18 ± 0.05	0.260	0.492	0.007
**Other subcortical structures**						
Nucleus accumbens	0.03 ± 0.01	0.03 ± 0.02	0.02 ± 0.02	0.358	0.594	0.001
Ventral DC	0.66 ± 0.06	0.63 ± 0.09	0.65 ± 0.08	0.592	0.686	0.000

DC: diencephalon; %TIV: percentage of total intracranial volume; *q*: Benjamini–Hochberg FDR-corrected *p* value across all volumetric and thickness variables; ε^2^: epsilon-squared effect size. Data are presented as mean ± SD. Groups were compared using the Kruskal–Wallis test with Bonferroni-adjusted Dunn post hoc comparisons; different superscript letters in the same row indicate significant pairwise differences. ‡ Omnibus *p* value was significant but no pairwise comparison remained significant after Bonferroni correction. * *p* < 0.05; † *q* < 0.05.

**Figure 2:**
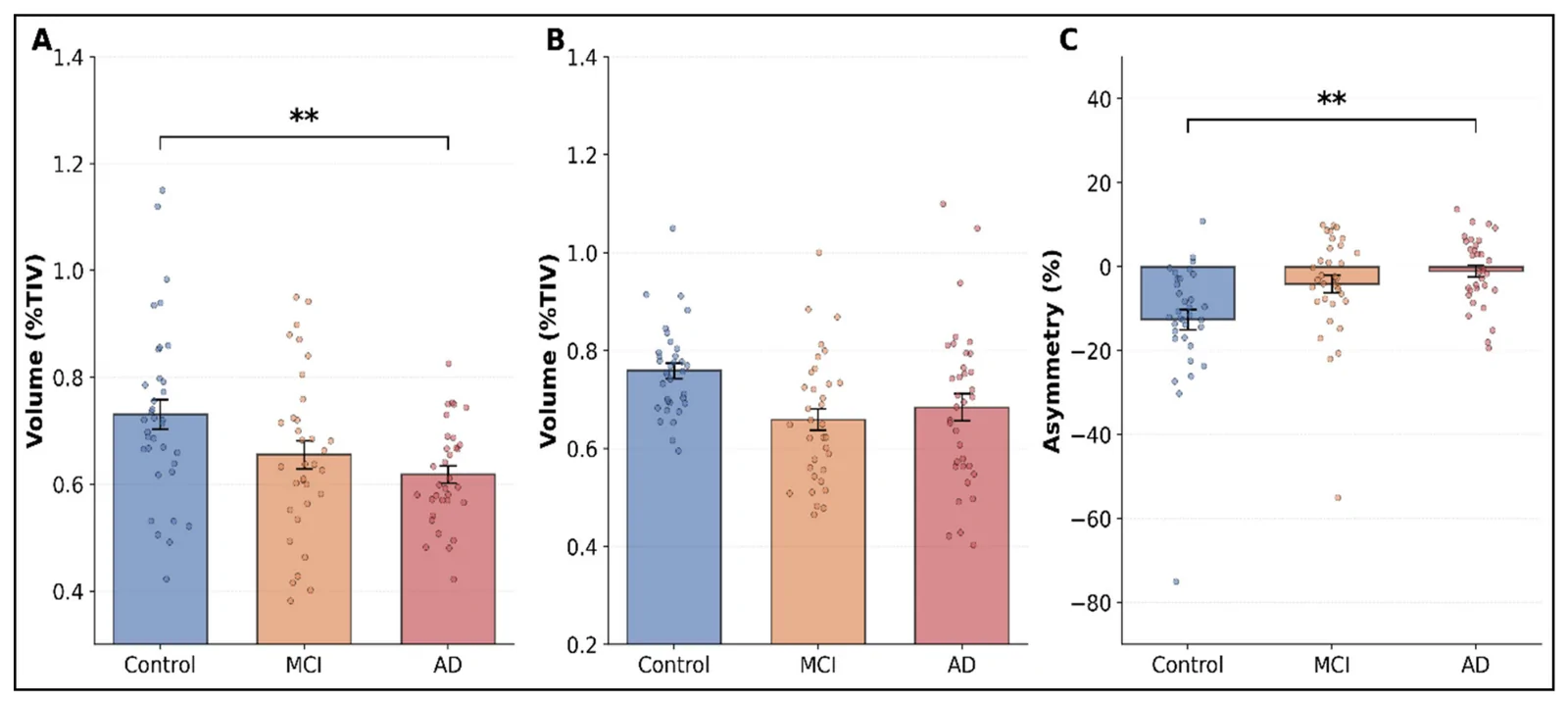
Comparison of regional brain volumetric measures across cognitive groups (HC, MCI, and AD/dementia): (**A**) anterior cingulate gyrus (ACgG) volume (%TIV), (**B**) middle cingulate gyrus (MCgG) volume (%TIV), and (**C**) insular lobe asymmetry (%). Box plots show the median and interquartile range with individual data points. Group comparisons were performed using the Kruskal–Wallis test with Bonferroni-adjusted Dunn post hoc comparisons. ACgG: anterior cingulate gyrus; MCgG: middle cingulate gyrus; %TIV: percentage of total intracranial volume; HC: healthy control; MCI: mild cognitive impairment; AD: Alzheimer’s disease. ** *p* < 0.001.

No significant group differences were observed in total frontal, temporal, parietal, occipital, limbic, or insular lobe volumes (Table 4). Moreover, mean cortical thickness measurements did not differ significantly between the groups. In contrast, significant differences were observed in several asymmetry indices. Parietal and insular lobe asymmetry indices were higher in the AD group than in the HC group (*p* = 0.004 and *p* = 0.011, respectively). Insular cortical thickness asymmetry was also higher in the AD group than in both the MCI and HC groups (*p* < 0.001). All three findings remained significant after FDR correction (*q* = 0.020, *q* = 0.049, and *q* = 0.004, respectively). Insular lobe asymmetry is further illustrated in Figure 2C.

**Table 4:** Lobar Volumes and Cortical Thickness by Cognitive Group.

Variables	AD (Mean ± SD)	MCI (Mean ± SD)	HC (Mean ± SD)	*p*	*q*	ε^2^
**Lobar volumes (%TIV)**						
Total frontal lobe	11.06 ± 1.60	11.51 ± 1.52	11.13 ± 2.52	0.533	0.681	0.000
Frontal lobe asymmetry (%)	4.15 ± 6.12	1.89 ± 6.34	2.52 ± 3.39	0.214	0.460	0.011
Total temporal lobe	6.59 ± 1.31	6.57 ± 1.20	6.99 ± 1.38	0.094	0.265	0.027
Temporal lobe asymmetry (%)	3.99 ± 8.81	2.96 ± 9.39	0.92 ± 5.51	0.074	0.225	0.032
Total parietal lobe	6.36 ± 1.09	6.58 ± 0.77	6.56 ± 1.38	0.541	0.681	0.000
Parietal lobe asymmetry (%)	4.13 ± 9.23 ᵃ	0.35 ± 7.80 ᵃᵇ	−0.40 ± 4.18 ᵇ	0.004 *	0.020 †	0.094
Total occipital lobe	4.73 ± 0.79	4.68 ± 0.85	4.46 ± 1.02	0.351	0.594	0.001
Occipital lobe asymmetry (%)	1.69 ± 9.82	−1.79 ± 8.64	−1.68 ± 7.09	0.063	0.203	0.036
Total limbic lobe	2.59 ± 0.50	2.58 ± 0.42	2.61 ± 0.77	0.434	0.669	0.000
Limbic lobe asymmetry (%)	0.02 ± 8.08	−1.75 ± 8.17	−2.77 ± 7.55	0.440	0.669	0.000
Total insular lobe	1.78 ± 0.29	1.86 ± 0.42	1.81 ± 0.64	0.651	0.731	0.000
Insular lobe asymmetry (%)	−0.42 ± 10.04 ᵃ	−6.08 ± 17.73 ᵃᵇ	−9.31 ± 16.20 ᵇ	0.011 *	0.049 †	0.071
**Cortical thickness (mm)**						
Frontal thickness	2.15 ± 0.72	2.19 ± 0.53	1.94 ± 0.72	0.308	0.536	0.004
Frontal thickness asymmetry (%)	5.02 ± 8.01	1.73 ± 7.45	2.66 ± 6.75	0.210	0.460	0.011
Temporal thickness	2.63 ± 0.76	2.64 ± 0.66	2.29 ± 0.87	0.198	0.451	0.013
Temporal thickness asymmetry (%)	1.76 ± 10.71	−2.29 ± 13.31	−1.40 ± 8.18	0.151	0.367	0.018
Parietal thickness	1.77 ± 0.61	1.82 ± 0.53	1.65 ± 0.63	0.436	0.669	0.000
Parietal thickness asymmetry (%)	2.38 ± 15.51	−0.21 ± 11.98	−0.49 ± 9.69	0.592	0.686	0.000
Occipital thickness	2.01 ± 0.65	1.91 ± 0.57	1.78 ± 0.74	0.139	0.363	0.020
Occipital thickness asymmetry (%)	−0.59 ± 10.99	−1.17 ± 12.54	−3.35 ± 7.78	0.473	0.681	0.000
Limbic thickness	2.55 ± 0.59	2.59 ± 0.57	2.49 ± 0.86	0.987	0.989	0.000
Limbic thickness asymmetry (%)	2.29 ± 9.73	−0.45 ± 7.13	1.06 ± 6.41	0.264	0.492	0.007
Insular thickness	2.10 ± 0.68	2.31 ± 0.66	2.09 ± 0.85	0.479	0.681	0.000
Insular thickness asymmetry (%)	11.32 ± 11.68 ᵃ	−0.57 ± 20.29 ᵇ	−0.06 ± 10.96 ᵇ	<0.001 *	0.004 †	0.140

AD: Alzheimer’s disease; MCI: mild cognitive impairment; HC: healthy control; %TIV: percentage of total intracranial volume; *q*: Benjamini–Hochberg FDR-corrected *p* value across all volumetric and thickness variables; ε^2^: epsilon-squared effect size. Volume measures are expressed as %TIV; cortical thickness measures are expressed in millimetres; asymmetry indices are expressed as percentages. Groups were compared using the Kruskal–Wallis test with Bonferroni-adjusted Dunn post hoc comparisons; different superscript letters in the same row indicate significant pairwise differences. Omnibus *p* value was significant but no pairwise comparison remained significant after Bonferroni correction. * *p* < 0.05; † *q* < 0.05.

No significant group differences were observed in the entorhinal cortex or in parahippocampal gyrus (PHG) and cortical thickness measurements (Table 5). The anterior cingulate gyrus (ACgG) volume differed significantly across groups (*p* = 0.004) and was lower in the AD group than in the HC group; this finding remained significant after FDR correction (*q* = 0.020). The middle cingulate gyrus (MCgG) volume also showed a significant overall group difference (*p* = 0.021). Post hoc analysis identified a significant MCI–HC difference (*p* = 0.037), whereas the AD–HC comparison did not reach statistical significance (*p* = 0.062). The MCgG finding did not remain significant after FDR correction (*q* = 0.076). Posterior cingulate gyrus (PCgG) volume and cortical thickness were comparable across groups. Moreover, anterior and posterior insular volumes did not differ significantly between the groups (*p* > 0.05). The ACgG and MCgG findings are illustrated in Figure 2A and Figure 2B, respectively.

**Table 5:** Volume, Cortical Thickness, and Asymmetry Measures in Cortical Regions.

Variables	AD (Mean ± SD)	MCI (Mean ± SD)	HC (Mean ± SD)	*p*	*q*	ε^2^
**Entorhinal cortex**						
Total entorhinal cortex (%TIV)	0.25 ± 0.06	0.25 ± 0.05	0.24 ± 0.07	0.947	0.987	0.000
Right entorhinal cortex (%TIV)	0.13 ± 0.04	0.13 ± 0.03	0.12 ± 0.04	0.989	0.989	0.000
Left entorhinal cortex (%TIV)	0.12 ± 0.03	0.12 ± 0.03	0.12 ± 0.03	0.509	0.681	0.000
Entorhinal asymmetry (%)	6.78 ± 15.92	6.81 ± 27.55	−1.11 ± 18.83	0.148	0.367	0.018
**Parahippocampal gyrus**						
Total PHG (%TIV)	0.36 ± 0.07	0.38 ± 0.06	0.37 ± 0.07	0.569	0.681	0.000
Right PHG (%TIV)	0.18 ± 0.04	0.19 ± 0.04	0.18 ± 0.04	0.565	0.681	0.000
Left PHG (%TIV)	0.18 ± 0.04	0.19 ± 0.04	0.19 ± 0.04	0.494	0.681	0.000
PHG asymmetry (%)	−3.77 ± 13.86	−1.47 ± 19.50	−2.88 ± 11.91	0.897	0.950	0.000
PHG cortical thickness (mm)	2.25 ± 0.91	2.14 ± 0.81	1.74 ± 1.05	0.078	0.227	0.031
**Cingulate gyrus**						
ACgG (%TIV)	0.61 ± 0.10 ᵃ	0.65 ± 0.15 ᵃᵇ	0.72 ± 0.14 ᵇ	0.004 *	0.020 †	0.092
ACgG cortical thickness (mm)	2.94 ± 0.64	3.06 ± 0.63	3.06 ± 1.00	0.245	0.492	0.008
MCgG (%TIV)	0.66 ± 0.12 ᵃᵇ	0.67 ± 0.15 ᵃ	0.73 ± 0.10 ᵇ	0.021 *	0.076	0.058
MCgG cortical thickness (mm)	2.21 ± 0.64	2.34 ± 0.64	2.37 ± 0.90	0.643	0.731	0.000
PCgG (%TIV)	0.63 ± 0.12	0.64 ± 0.13	0.66 ± 0.09	0.675	0.735	0.000
PCgG cortical thickness (mm)	2.61 ± 0.69	2.57 ± 0.73	2.49 ± 0.83	0.898	0.950	0.000
**Insular cortex**						
Anterior insula (AIns, %TIV)	0.51 ± 0.09	0.56 ± 0.13	0.52 ± 0.16	0.250	0.492	0.008
Posterior insula (PIns, %TIV)	0.28 ± 0.07	0.29 ± 0.10	0.26 ± 0.09	0.965	0.989	0.000

AD: Alzheimer’s disease; MCI: mild cognitive impairment; HC: healthy control; %TIV: percentage of total intracranial volume; PHG: parahippocampal gyrus; ACgG: anterior cingulate gyrus; MCgG: middle cingulate gyrus; PCgG: posterior cingulate gyrus; AIns: anterior insula; PIns: posterior insula; *q*: Benjamini–Hochberg FDR-corrected *p* value across all volumetric and thickness variables; ε^2^: epsilon-squared effect size. Volume measures are expressed as %TIV; cortical thickness measures are expressed in millimetres; asymmetry indices are expressed as percentages. Groups were compared using the Kruskal–Wallis test with Bonferroni-adjusted Dunn post hoc comparisons; different superscript letters in the same row indicate significant pairwise differences. * *p* < 0.05; † *q* < 0.05.

**Figure 3:**
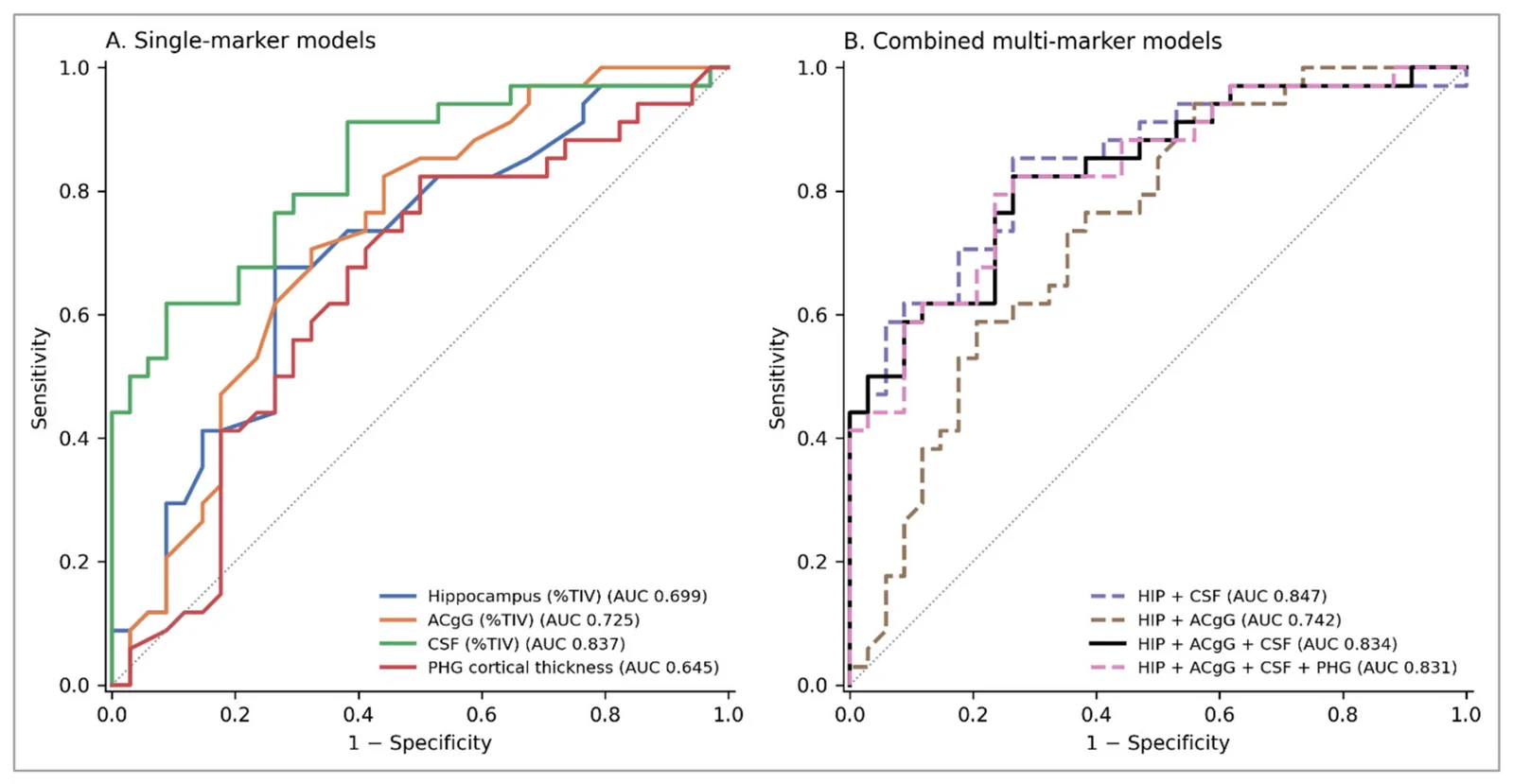
ROC curves showing the diagnostic performance of MRI volumetric markers in differentiating the HC group (*n* = 34) from the AD group (*n* = 34). (**A**) Single-marker models: CSF (%TIV), ACgG (%TIV), hippocampus (%TIV) and PHG cortical thickness. (**B**) Combined multi-marker models generated using logistic regression. The dashed diagonal line represents the reference line (AUC = 0.50). %TIV: percentage value normalized to total intracranial volume; ACgG: anterior cingulate gyrus; PHG: parahippocampal gyrus; CSF: cerebrospinal fluid; HC: healthy control; AD: Alzheimer’s disease.

**Figure 4:**
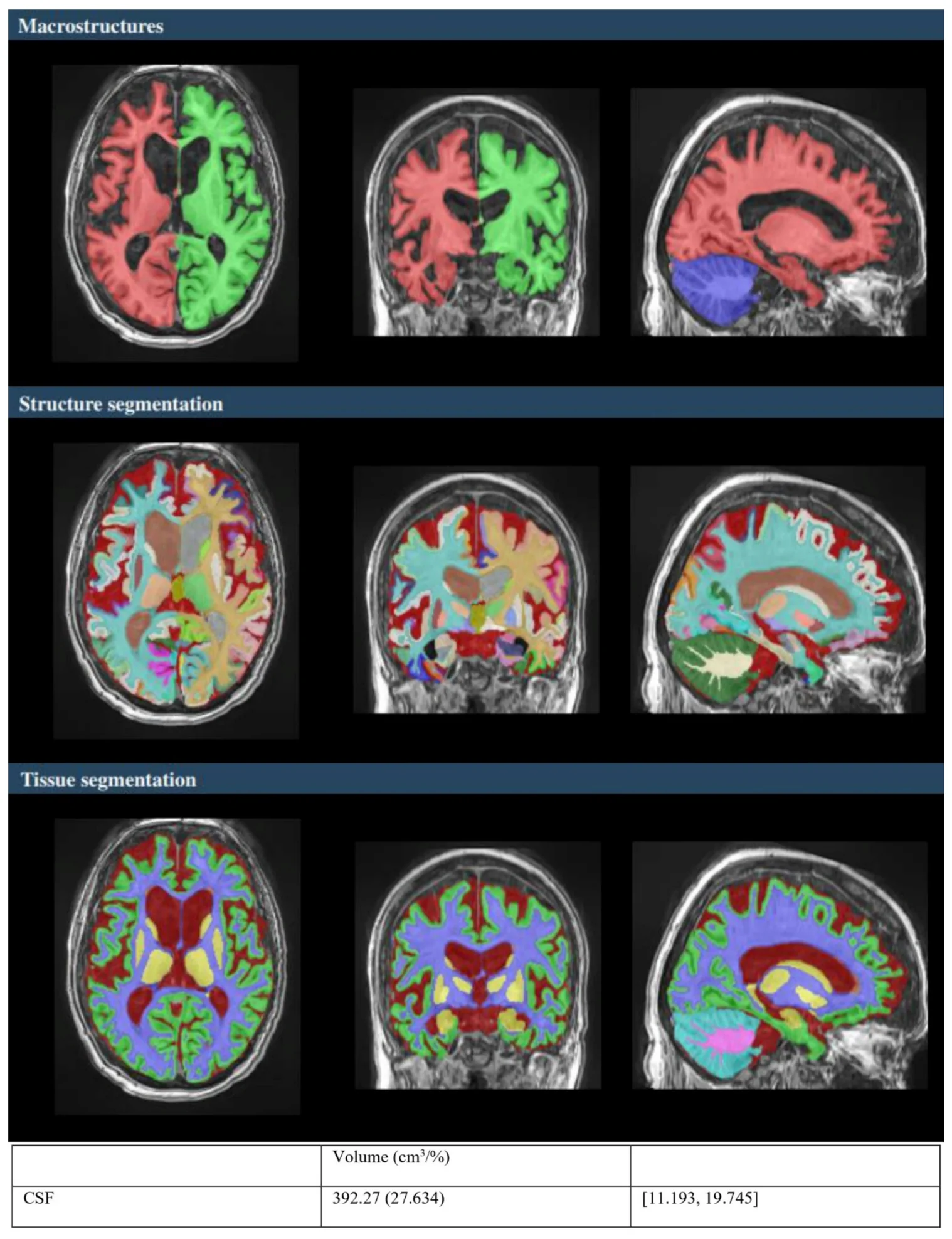
Representative volBrain cerebrospinal fluid (CSF) segmentation output from an anonymized AD patient, illustrating the CSF measure that provided the highest single-marker discrimination in Table 6. In the tissue segmentation images, CSF is shown in dark red, gray matter in purple, and white matter in green.

ROC analysis was performed to evaluate the discriminative performance of four prespecified volumetric markers for differentiating AD from HC (Table 6). The markers had been selected before examination of the between-group results on the basis of previous volumetric and neuropathological studies of AD; no data-driven or stepwise selection procedure was used. Among the single-marker models, CSF volume showed the highest performance (AUC = 0.837, 95% CI 0.728–0.920, *p* < 0.001), followed by ACgG volume (AUC = 0.725), hippocampal volume (AUC = 0.699), and PHG cortical thickness (AUC = 0.645). Among the combined logistic regression models, the highest performance was obtained with hippocampus + CSF model (AUC = 0.847; 95% CI 0.743–0.930). However, no combined model was statistically superior to CSF volume alone on DeLong testing (ΔAUC −0.095 to +0.010; all *p* > 0.13), and bootstrap optimism correction and cross-validation indicated limited overfitting (Supplementary Material S2; Figure 3). A representative volBrain output showing CSF segmentation and volume measurement in an anonymized patient with AD is presented in Figure 4.

**Figure 5:**
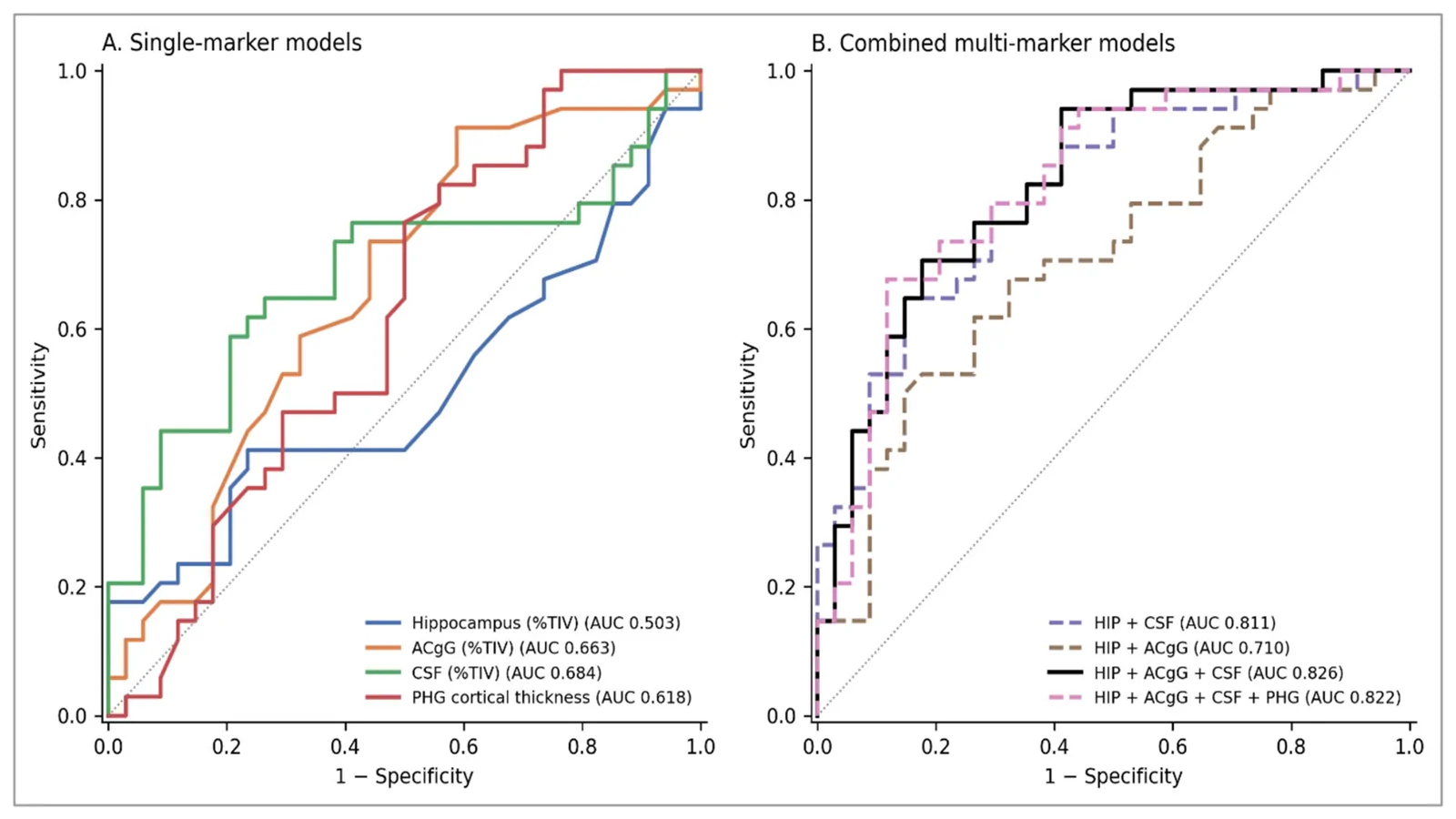
ROC curves of MRI volumetric markers for differentiating HC (*n* = 34) from MCI (*n* = 34). (**A**) Single-marker models: CSF (%TIV), ACgG (%TIV), hippocampus (%TIV), and PHG cortical thickness. (**B**) Combined multi-marker models. The dotted diagonal line represents the reference line (AUC = 0.50). %TIV: percentage of total intracranial volume; ACgG: anterior cingulate gyrus; PHG: parahippocampal gyrus; CSF: cerebrospinal fluid; HC: healthy control; MCI: mild cognitive impairment.

**Figure 6:**
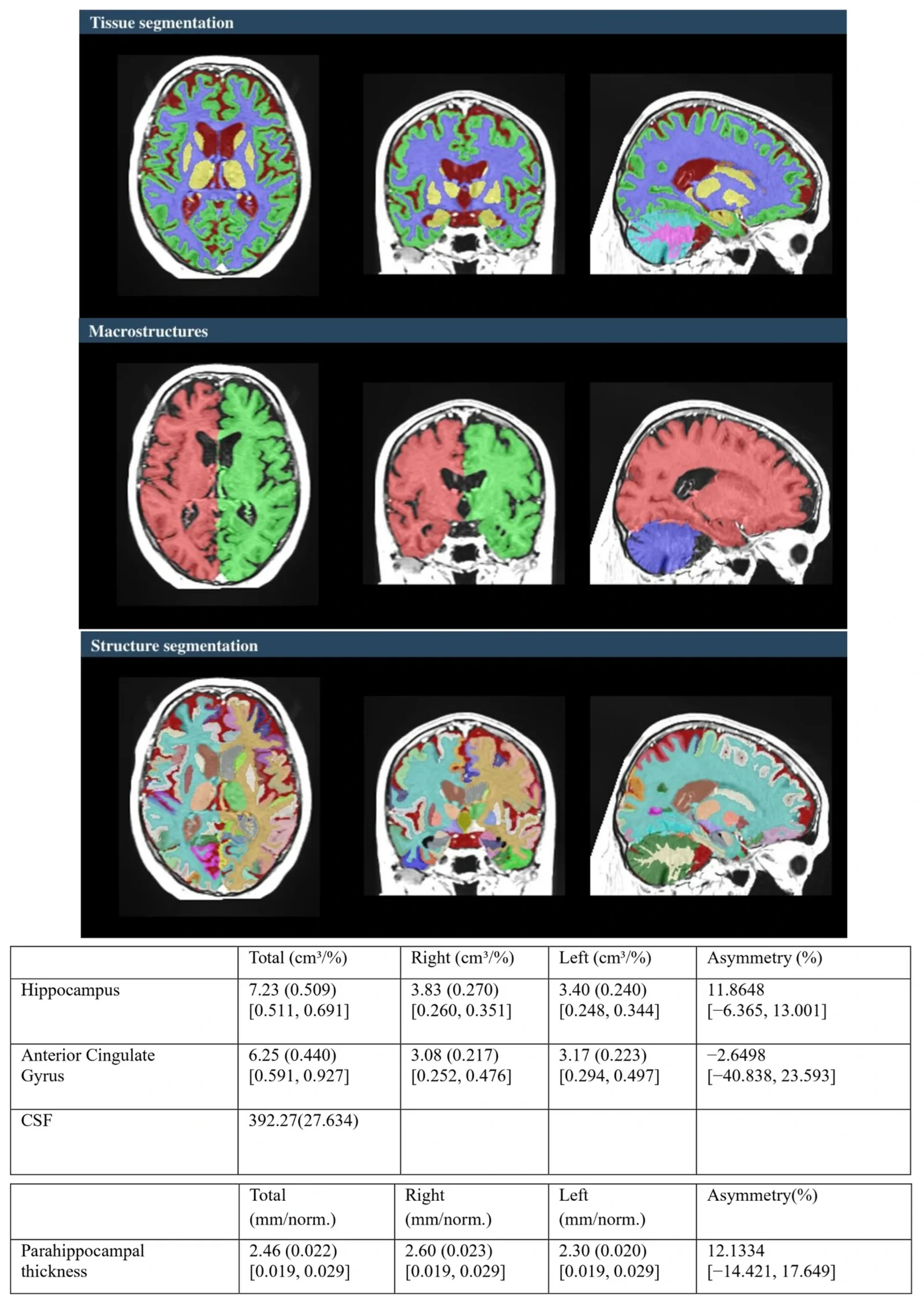
Representative examples of automated MRI volumetric segmentation from an anonymized MCI patient. The upper panel shows tissue segmentation, with cerebrospinal fluid (CSF) displayed in dark red, gray matter in purple, and white matter in green. The middle panel illustrates macrostructure segmentation, including the cerebral hemispheres and cerebellum. The lower panel shows automated segmentation of individual cortical and subcortical structures. The accompanying tables present representative normalized volumetric measurements.

ROC analysis showed limited single-marker performance for differentiating MCI from HC (Table 7). Among the single-marker models, CSF volume showed the highest discriminative performance (AUC = 0.684, 95% CI 0.553–0.811, *p* = 0.004), followed by ACgG volume (AUC = 0.663) and PHG cortical thickness (AUC = 0.618). Hippocampal volume showed no discriminative value for MCI–HC differentiation (AUC = 0.503, *p* = 0.966). Multi-marker logistic regression models yielded higher AUCs than the individual markers. The hippocampus + ACgG + CSF model showed the highest AUC (0.826, 95% CI 0.720–0.914), followed by the four-marker model including PHG cortical thickness (AUC = 0.822) and the hippocampus + CSF model (AUC = 0.811). According to DeLong’s test, the hippocampus + ACgG + CSF model significantly outperformed CSF volume alone (ΔAUC = 0.142, *p* = 0.026). The combined models retained higher internally validated AUC estimates after bootstrap optimism correction and repeated cross-validation. However, the events-per-variable ratio of 8.5 for the four-marker model indicates that it should be considered exploratory (Supplementary Material S2; Figure 5). A representative volBrain output from an anonymized patient with MCI, showing the hippocampal, ACgG, and CSF volumes and PHG cortical thickness used in the MCI–HC diagnostic models, is presented in Figure 6.

**Figure 7:**
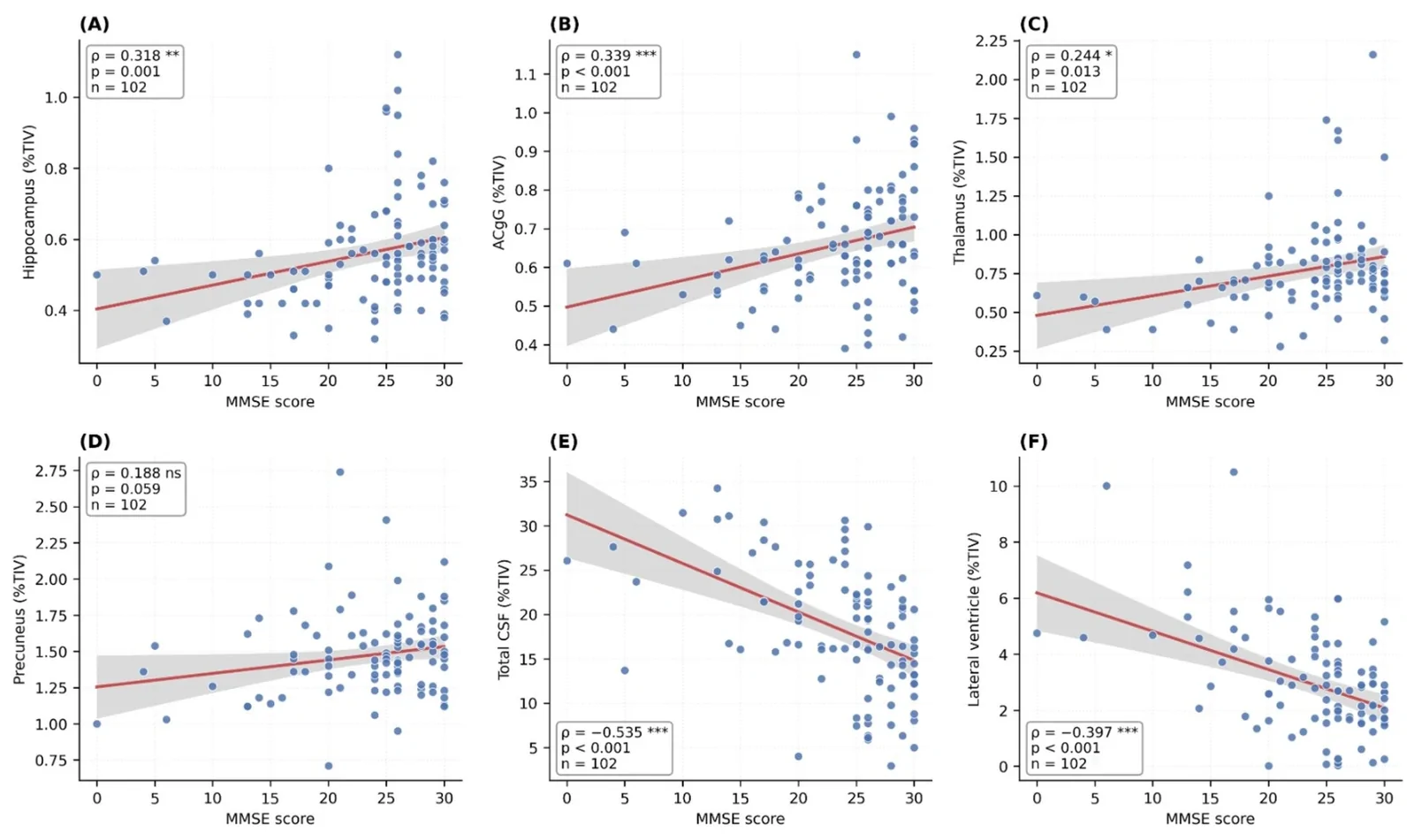
Relationship between MMSE score and key brain regions in the entire cohort. Positive correlations: (**A**) hippocampus, (**B**) anterior cingulate gyrus, (**C**) thalamus, and (**D**) precuneus. Negative correlations: (**E**) total CSF and (**F**) lateral ventricle. The red line indicates the linear regression fit, and the gray band represents the 95% confidence interval. Spearman’s ρ, the corresponding *p* value, and the sample size are presented in each panel. Statistical significance is denoted as follows: * *p* < 0.05, ** *p* < 0.01, *** *p* < 0.001, ns: not statistically significant (*p* ≥ 0.05). MMSE: Mini-Mental State Examination, ACgG: anterior cingulate gyrus, CSF: cerebrospinal fluid, %TIV: percentage of total intracranial volume.

Given that MMSE scores contributed to diagnostic group classification, pooled correlations were not fully independent of between-group differences. Therefore, group-adjusted partial correlations and separate within-group correlations were also reported (Supplementary Material S3). In the pooled analysis, MMSE score correlated with total CSF volume (ρ = −0.54), LV volume (ρ = −0.40), ACgG volume (ρ = +0.34), hippocampal volume (ρ = +0.32), thalamic volume (ρ = +0.24), and precuneus volume (ρ = +0.19) (Figure 7). After adjustment for group, only total CSF volume (partial ρ = −0.31; *p* = 0.002), LV volume (ρ = −0.23; *p* = 0.019), and hippocampal volume (ρ = +0.23; *p* = 0.023) remained significant, whereas the associations for ACgG, thalamus, and precuneus lost significance. Within-group analyses showed that these associations persisted in the AD and MCI groups but were absent in controls (Supplementary Material S3). This pattern suggests that the associations of CSF and hippocampal volumes with MMSE are not solely explained by between-group differences, whereas the associations for ACgG, thalamus, and precuneus appear to be largely influenced by diagnostic group membership.

Associations of regional volumes with age, education, smoking exposure, sex, and alcohol use are presented in Table 8. Before FDR correction, hippocampal volume was positively associated with education (ρ = 0.198, *p* = 0.046) and was higher in men than in women (*p* = 0.009), while total CSF volume was positively associated with age (ρ = 0.201, *p* = 0.042). None of these findings remained significant after Benjamini–Hochberg correction across the 30 tests (all *q* ≥ 0.268) and were therefore considered exploratory. Alcohol use was dichotomized as any use (*n* = 13) versus no use (*n* = 89) and analysed using exact permutation testing because the presence of only one current drinker precluded a valid three-category comparison; no association with alcohol use was significant (all *p* ≥ 0.05). No other significant associations were observed.

**Table 8:** Associations Between Regional Brain Volumes and Age, Education, Smoking, Sex, and Alcohol Use.

Variables (%TIV)	Age ρ (*p*; *q*)	Education ρ (*p*; *q*)	Smoking (Pack-Year) ρ (*p*; *q*)	Sex *p* (F vs. M); *q*	Alcohol Use *p*; *q*
**Hippocampus**	−0.007 (0.948; 0.948)	+0.198 (0.046; 0.459)	+0.166 (0.095; 0.474)	0.009; 0.268 (M > F)	0.923; 0.948
**ACgG**	+0.079 (0.431; 0.708)	+0.132 (0.185; 0.555)	−0.012 (0.908; 0.948)	0.386; 0.708	0.506; 0.708
**Thalamus**	+0.061 (0.542; 0.708)	−0.105 (0.293; 0.692)	+0.061 (0.543; 0.708)	0.422; 0.708	0.933; 0.948
**Precuneus**	+0.157 (0.114; 0.477)	+0.035 (0.730; 0.876)	+0.094 (0.345; 0.708)	0.203; 0.555	0.075; 0.474
**Total CSF**	+0.201 (0.042; 0.459)	−0.152 (0.127; 0.477)	−0.169 (0.089; 0.474)	0.186; 0.555	0.471; 0.708
**LV**	+0.104 (0.300; 0.692)	−0.087 (0.387; 0.708)	−0.067 (0.505; 0.708)	0.625; 0.781	0.886; 0.948

F: female; M: male; TIV: total intracranial volume; ACgG: anterior cingulate gyrus; CSF: cerebrospinal fluid; LV: lateral ventricle. Associations with age, education, and smoking exposure are presented as Spearman’s ρ with corresponding *p*-values. Sex and alcohol-use comparisons were performed using the Mann–Whitney U test and Kruskal–Wallis test, respectively. All volume measures are expressed as %TIV. Sex groups included 51 women and 51 men; alcohol-use groups included non-users (*n* = 89), social drinkers (*n* = 12), and current drinkers (*n* = 1). No association remained significant after FDR correction; uncorrected associations are therefore considered exploratory. *p* < 0.05.

## 4. Discussion

In the present study, total, cortical, and subcortical brain volumes obtained using automated MRI volumetry were compared between participants categorized into AD, MCI, and HC groups, and their associations with cognitive performance were evaluated. The findings demonstrated lower total WM, subcortical GM, and total cerebrum volumes, along with higher CSF volume, in the AD group. In contrast, the lobar cortical volumes and cortical thickness measurements did not differ significantly between the groups in this sample. In the ROC analyses, CSF volume showed the highest single-marker discriminative performance for differentiating AD from HC, whereas combined volumetric models provided better discrimination between MCI and HC. Overall, our findings suggest that automated MRI volumetry may provide complementary quantitative information on structural brain changes associated with clinically defined AD and MCI.

### 4.1. Total White Matter Loss and Reduced Cerebral Volume

One of the main findings of our study was that total WM and NAWM volumes were lower in the AD and MCI groups than in HC. In contrast, no significant difference was observed between groups in AbAWM volume. This suggests that the reduction in total WM volume was mainly related to lower NAWM volume rather than to a greater burden of visibly abnormal white matter. Previous diffusion tensor imaging studies have reported that microstructural WM damage may occur in AD and MCI, even in the absence of visible lesions [21,22]. Total cerebrum volume was also lowest in the AD group, consistent with more extensive global parenchymal loss. This is in line with previous studies showing that structural changes in AD are not limited to the hippocampus and may also involve thalamic, striatal, cingulate, parietal, and insular regions [7,23].

No significant group differences were observed in cerebellar or brainstem volumes, indicating no prominent macrostructural volume loss in these regions in our sample. However, the absence of volumetric differences should not be interpreted as indicating that the cerebellum and brainstem are unaffected by AD. Recent studies have shown that the cerebellum contributes to cognitive processes and that cortico-cerebellar functional connectivity may be disrupted in patients with AD and MCI [24,25]. Similarly, neuropathological studies have suggested that tau pathology may begin in the lower brainstem nuclei, particularly the locus coeruleus and related monoaminergic systems, before involving the transentorhinal cortex in some cases [26]. Therefore, the cerebellar and brainstem findings in our study suggest not the complete preservation of these regions but rather the absence of prominent volume loss at the macrovolumetric level.

In parallel with the reduction in WM volume, our study demonstrated enlargement of CSF compartments. The total CSF, external CSF, and LV volumes were highest in the AD group and lowest in the HC group, while the MCI group showed intermediate values for most measures. Total CSF volume also showed the highest single-marker discriminative performance for AD–HC differentiation in our cohort. Increased total and external CSF volumes may reflect compensatory expansion accompanying the loss of brain parenchymal volume, whereas increased LV volume may indicate central atrophy and periventricular tissue loss. In the literature, ventricular enlargement has been regarded as an indirect but sensitive MRI marker of neuronal loss and brain atrophy in AD and MCI [27]. More recent studies have also associated baseline ventricular volume with cognitive status and clinical progression in AD [28], and inferior LV enlargement with conversion from MCI to AD [29]. Our findings support the concept that parenchymal atrophy and CSF expansion progress together. Nevertheless, because ventricular enlargement may also be influenced by aging, vascular changes, and global brain atrophy, it should not be interpreted as an AD-specific marker in isolation but rather evaluated together with hippocampal, subcortical, and global volume measures.

### 4.2. Subcortical GM Atrophy: Hippocampus, Thalamus, and Striatum

Volumetric analysis of subcortical structures showed that the most prominent group differences were observed in the hippocampus, thalamus, caudate nucleus, and putamen. Regarding the hippocampal complex, total, right, and left hippocampal volumes were significantly lower in the AD group than in both the MCI and HC groups, whereas no significant differences were observed between the MCI and HC groups. This finding is consistent with the extensive literature linking hippocampal atrophy to AD-related neuropathology and supporting its diagnostic relevance and association with disease severity [30,31]. Previous studies have also reported that hippocampal atrophy in AD is generally bilateral, although the extent of right- and left-sided involvement may vary according to anatomical characteristics, disease stage, and the hippocampal subfields evaluated [32,33]. In contrast to reports of variable hemispheric involvement, hippocampal asymmetry did not differ between the groups, suggesting no clear lateralization pattern in our cohort.

The absence of a significant hippocampal volume difference between the MCI and HC groups may reflect the clinical and biological heterogeneity of MCI rather than the absence of medial temporal involvement. MCI includes amnestic and non-amnestic subtypes with different cognitive profiles and patterns of brain involvement. In amnestic MCI, memory impairment predominates and greater involvement of the hippocampus and medial temporal lobe is expected, whereas in non-amnestic MCI, non-memory domains such as attention, executive function, language, or visuospatial abilities may be affected, reflecting involvement of different cortical–subcortical networks [34]. A recent study reported that structural network alterations involving the hippocampus, parahippocampal gyrus, amygdala, and temporal lobe connections varied among individuals with MCI, and that the subtype characterized by more prominent medial temporal–limbic involvement was associated with faster cognitive decline and progression to AD [35]. The cross-sectional design should also be considered, as hippocampal atrophy may differ in timing and severity across individuals and may not be detectable at a single time point. Longitudinal studies have further suggested that changes in hippocampal volume over time may be more informative than a single cross-sectional measurement for predicting progression from MCI to AD [29]. In addition, the absence of amyloid and tau biomarker confirmation limited our ability to relate these structural findings to specific biological subgroups. Therefore, the lack of a significant MCI–HC difference may reflect both the heterogeneity of MCI and the limited sensitivity of cross-sectional assessment rather than the absence of hippocampal involvement.

Thalamic volumes differed significantly between the groups. Total and left thalamic volumes were lower in the AD group than in both the MCI and HC groups, whereas right thalamic volume differed mainly between the AD and MCI groups. This pattern suggests that thalamic involvement was present in AD, although the distribution of group differences was not uniform across hemispheres. Similarly, caudate nucleus and putamen volumes were significantly lower in the AD group than in the MCI group, whereas no significant difference was observed between the AD and HC groups. The relatively higher striatal volumes observed in the MCI group may reflect the clinical and biological heterogeneity of MCI, within-group variability, the limited sample size, or measurement effects related to TIV normalization.

The thalamus plays an important role in memory, attention, arousal, and executive functions through its thalamocortical and thalamo-limbic connections with the hippocampus, cingulate gyrus, and prefrontal cortex. The caudate nucleus and putamen are also involved in executive functions, cognitive flexibility, processing speed, and motor–cognitive integration [36,37]. Previous studies have identified the anterior thalamic nuclei as key components of the episodic memory network [38] and have suggested that thalamic and striatal involvement may become more prominent as AD-related structural changes extend beyond the medial temporal lobe [23]. Longitudinal MRI studies have also shown that thalamic volume loss may be associated with progression to AD [39]. However, given the cross-sectional design of our study, the present findings should not be interpreted as evidence of temporal progression. Overall, the thalamic, caudate, and putaminal findings support the view that AD-related neurodegeneration may involve broader thalamo-limbic and cortico-subcortical networks beyond the medial temporal structures.

The absence of significant group differences in amygdala, pallidum, nucleus accumbens, and ventral DC volumes suggests that subcortical involvement does not occur to the same extent across all structures. Although amygdalar atrophy is frequently reported in AD [2], the small size of this structure, its susceptibility to automated segmentation variability, within-group volumetric heterogeneity, and disease-stage-related differences may explain the lack of a significant group difference in our study. Moreover, amygdalar involvement may be more variable than hippocampal involvement. Consistent with this, a study reported widespread volume reductions in hippocampal subfields in AD, amnestic MCI, and HC groups, whereas no significant volume loss was observed in amygdalar subfields after correction for multiple comparisons [40].

Basal forebrain volume showed a significant overall group effect, although no pairwise comparison remained significant after correction; therefore, this finding should be interpreted cautiously. The cholinergic basal forebrain is considered an important site of early involvement in AD. A recent study reported lower cholinergic basal forebrain volumes in patients with AD than in HC and found that this reduction was associated with cortical cholinergic denervation [41]. However, volumetric assessment of small structures with complex anatomical boundaries, such as the basal forebrain, may be influenced by segmentation methods, subregional definitions, image quality, and sample size. Therefore, the absence of significant pairwise differences in our study may reflect methodological limitations and within-group variability rather than a lack of cholinergic system involvement.

### 4.3. Lobar Volumes, Parietal Asymmetry, and Insular Cortical Thickness Asymmetry

When cortical and lobar measures were evaluated, no significant group differences were observed in total frontal, temporal, parietal, occipital, limbic, or insular lobe volumes, or in mean cortical thickness. This suggests that total lobar volume measurements may be less sensitive than subcortical volumes and CSF measurements for differentiating AD, MCI, and HC groups in our sample. In contrast, the marked differences in parietal lobe asymmetry, insular lobe asymmetry, and insular cortical thickness asymmetry in the AD group are noteworthy. This finding suggests that interhemispheric balance may be altered in specific cortical regions, even in the absence of significant changes in total lobar volume, and that asymmetry measures may provide additional information beyond conventional volumetric measures.

A large longitudinal lifespan study showed that cortical thickness asymmetry changes with aging and that these asymmetry alterations may be accelerated in AD. Asymmetry changes were particularly evident in heteromodal cortical regions involved in higher-order cognitive networks, including the insular and lateral temporoparietal cortices [10]. Consistent with these observations, our study demonstrated significant parietal and insular asymmetry despite the absence of differences in total lobar volumes, suggesting that regional asymmetry measures may detect structural alterations not captured by conventional volumetric measures.

### 4.4. Cingulate Gyrus Alterations and Diagnostic Relevance

Among the cortical regions evaluated, the ACgG finding was particularly noteworthy. Although no significant group differences were observed in entorhinal cortex or PHG measurements, ACgG volume was lower in the AD group than in HC and remained significant after FDR correction. In contrast, the MCgG finding was less robust, as post hoc significance was limited to the MCI–HC comparison and the overall finding did not survive FDR correction.

The cingulate cortex is an important component of cortico-limbic networks involved in attention, executive function, cognitive control, motivation, and memory processes. In particular, the ACgG, together with the anterior insula, is considered a key node of the salience network and plays a role in cognitive control and attentional processes [42]. ACgG alterations across the AD spectrum have also been reported to be associated with cognitive function [43]. In our study, the lower ACgG volume observed in the AD group is consistent with previous evidence suggesting that AD-related structural changes extend beyond the medial temporal lobe [44]. Although ACgG volume was associated with cognitive performance in the pooled analysis, this relationship was attenuated after adjustment for diagnostic group, indicating that it was partly influenced by between-group differences. Nevertheless, ACgG volume showed moderate discriminative performance as an individual marker and may provide complementary information alongside hippocampal and CSF measures.

PHG cortical thickness showed limited standalone discriminative performance in our study. Although recent studies have demonstrated that medial temporal lobe subregions are affected to varying degrees in AD and MCI, and that the parahippocampal region may exhibit structural and functional alterations [45], adding PHG cortical thickness did not improve the performance of the best multi-marker model in our cohort. Previous studies have also suggested that individual structural MRI measures may have limited value when used alone and may be more informative when interpreted together with longitudinal, cognitive, and biomarker data [46]. Therefore, PHG cortical thickness should be regarded as a supportive morphometric measure of medial temporal network involvement rather than an independent diagnostic marker.

### 4.5. Diagnostic and Clinical Value of Combined MRI Volumetric Models

In the ROC analyses, CSF volume showed the strongest single-marker discriminative performance for AD–HC differentiation. Adding hippocampal or ACgG measures provided only limited additional benefit, and no combined model significantly outperformed CSF volume alone. This suggests that, in clinically defined AD, CSF-space enlargement accompanying global parenchymal loss may be one of the dominant structural features distinguishing patients from cognitively unimpaired controls.

In contrast, combined volumetric models performed better than individual markers for MCI–HC differentiation. The highest AUC was obtained with the hippocampus + ACgG + CSF model, whereas adding PHG cortical thickness did not further improve performance. This pattern suggests that the subtle and heterogeneous structural changes observed in MCI may be better captured by combining complementary regional and global measures rather than relying on a single marker. Given the modest, single-center sample and lack of external validation, these models should be considered exploratory and require independent validation, standardization, and evaluation across different populations before clinical application [47].

The associations between cognitive performance and volumetric measures further support the potential clinical relevance of automated MRI volumetry. In the pooled analysis, hippocampal, ACgG, thalamic, and precuneus volumes were positively associated with cognitive performance, whereas total CSF and LV volumes were negatively associated. After adjustment for diagnostic group, however, only hippocampal, total CSF, and LV volumes remained significantly associated with cognitive performance. This suggests that these associations were not solely explained by differences between the diagnostic groups, whereas the pooled associations involving ACgG, thalamus, and precuneus were partly influenced by group separation. These findings are consistent with previous evidence linking structural MRI measures to cognitive decline and support the value of longitudinal and multimodal approaches for prognostic assessment [46].

The main strengths of our study include the comparable sample sizes across the AD, MCI, and HC groups; the comprehensive assessment of cortical, subcortical, CSF, and hemispheric asymmetry measures using automated volumetry; and the use of TIV-normalized volumes. Automated segmentation may improve reproducibility and standardization while reducing observer-dependent variability.

Several limitations should also be acknowledged. First, the cross-sectional design does not allow causal inference or assessment of the rate of disease progression. Longitudinal follow-up data could more robustly demonstrate the diagnostic and prognostic value of volumetric change over time. The relatively limited sample size may have reduced statistical power, particularly for analyses of small-volume structures and so some regional differences may not have reached statistical significance. In addition, because amyloid or tau PET imaging and CSF biomarkers were not assessed, the direct relationship between MRI volumetric findings and AD pathology could not be confirmed. Accordingly, the AD and MCI groups represent clinically defined rather than biologically confirmed categories, and biological heterogeneity, particularly within the MCI group, cannot be excluded. Finally, the single-center referral-based sample and the absence of an independent external validation cohort may limit the generalizability of the findings and the clinical applicability of the diagnostic models.

## 5. Conclusions

This study suggests that automated MRI volumetry may provide complementary quantitative information on structural brain changes in clinically defined AD and MCI. Hippocampal atrophy remained a prominent regional finding, whereas CSF expansion showed the strongest single-marker discrimination for AD, and combined regional and global volumetric measures improved discrimination between the MCI and HC groups. These findings suggest that different volumetric markers may provide complementary information at different stages of cognitive impairment. Further multicenter, longitudinal, biomarker-characterized studies with independent validation are needed to determine the clinical utility of automated MRI volumetry.

## Figures and Tables

**Table 6:** Diagnostic Performance of MRI Volumetric Markers and Combined Models for AD–HC Differentiation.

Model	AUC	95% CI	*p*	Sensitivity (%)	Specificity (%)
Hippocampus (%TIV)	0.699	0.568–0.821	0.002	67.6	73.5
ACgG (%TIV)	0.725	0.593–0.838	<0.001	70.6	67.6
CSF (%TIV)	0.837	0.728–0.920	<0.001	61.8	91.2
PHG cortical thickness (mm)	0.645	0.507–0.778	0.030	82.4	50.0
Hippocampus + CSF	0.847	0.743–0.930	<0.001	85.3	73.5
Hippocampus + ACgG	0.742	0.606–0.855	<0.001	58.8	79.4
Hippocampus + ACgG + CSF	0.834	0.740–0.914	<0.001	82.4	73.5
Hippocampus + ACgG + CSF + PHG cortical thickness	0.831	0.730–0.918	<0.001	79.4	76.5

ACgG: anterior cingulate gyrus; PHG: parahippocampal gyrus; CSF: cerebrospinal fluid; AUC: area under the curve; CI: confidence interval; ; Optimal cut-off values were determined using the Youden index; combined models were generated by binary logistic regression.

**Table 7:** Diagnostic Performance of MRI Volumetric Markers and Combined Models for MCI–HC Differentiation.

Model	AUC	95% CI	*p*	Sensitivity (%)	Specificity (%)
Hippocampus (%TIV)	0.503	0.349–0.644	0.966	17.6	100.0
ACgG (%TIV)	0.663	0.522–0.783	0.014	91.2	41.2
CSF (%TIV)	0.684	0.553–0.811	0.004	58.8	79.4
PHG cortical thickness (mm)	0.618	0.477–0.747	0.083	76.5	50.0
Hippocampus + CSF	0.811	0.701–0.905	<0.001	64.7	85.3
Hippocampus + ACgG	0.710	0.586–0.833	<0.001	61.8	73.5
Hippocampus + ACgG + CSF	0.826	0.720–0.914	<0.001	70.6	82.4
Hippocampus + ACgG + CSF + PHG cortical thickness	0.822	0.715–0.908	<0.001	67.6	88.2

ACgG: anterior cingulate gyrus; PHG: parahippocampal gyrus; CSF: cerebrospinal fluid; AUC: area under the curve; CI: confidence interval; %TIV: percentage of total intracranial volume. AUC values were derived from binary logistic regression models using predicted probabilities as the test variable. Optimal cut-off values were determined using the Youden index. *p* < 0.05.

## Data Availability

The original contributions presented in this study are included in the article. Further inquiries can be directed to the corresponding author.

## Supplementary Materials

The following supporting information can be downloaded at: https://www.mdpi.com/article/10.3390/diagnostics16162596/s1. Supplementary Material S1: Representative complete automated volBrain volumetry report from a healthy control participant. Supplementary Material S2: Representative volBrain cerebrospinal fluid segmentation output from an Alzheimer’s disease patient. Supplementary Material S3: Representative volBrain regional volumetric and cortical thickness output from a mild cognitive impairment patient.

## Abbreviations

AbAWM	Abnormal Appearing White Matter
ACgG	Anterior Cingulate Gyrus
AD	Alzheimer’s Disease
AI	Asymmetry Index
AUC	Area Under the Curve
BMI	Body Mass Index
CSF	Cerebrospinal Fluid
DC	Diencephalon
GM	Grey Matter
HC	Healthy Control
LV	Lateral Ventricle
MCI	Mild Cognitive Impairment
MCgG	Middle Cingulate Gyrus
MMSE	Mini-Mental State Examination
MRI	Magnetic Resonance Imaging
NAWM	Normal Appearing White Matter
PHG	Parahippocampal Gyrus
ROC	Receiver Operating Characteristic
TIV	Total Intracranial Volume
WM	White Matter
