# Supplementary material for: Magnetic Resonance Imaging-Based Cortical and Subcortical Volumetric Changes in Alzheimer’s Disease: Association with Clinical Severity
Source: Diagnostics (Basel). 2026 Aug 16;16(16):2596. doi: 10.3390/diagnostics16162596 (PMC13511431; doi:10.3390/diagnostics16162596)
Supplement: Supplementary file 1 [file diagnostics-16-02596-s001.zip › Supplementary Material S1.pdf]

Supplementary Material S1: Representative complete automated volBrain volumetry report from a healthy control participant.

vol2Brain volumetry report

version 1.0 release 23-11-2021.

Subject: job1687612

**Sex**	
Male	
**Age**	
67	
**Report date**	
13-May-2024	
**Image orientation**	
radiological	
**Scale factor**	
0.71	
**SNR**	
149.80	

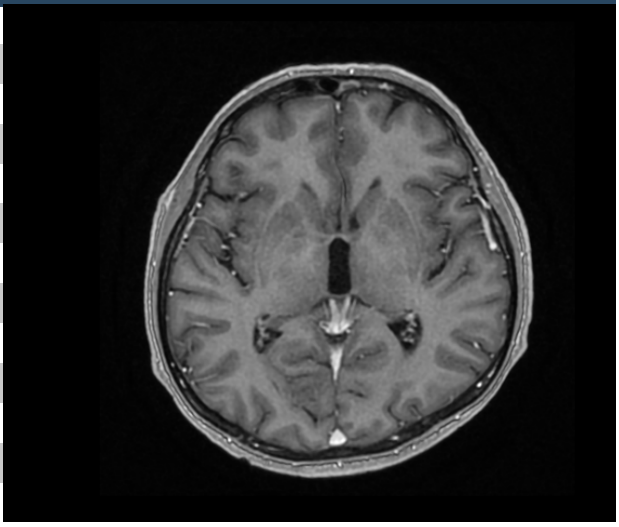

Tissue segmentation

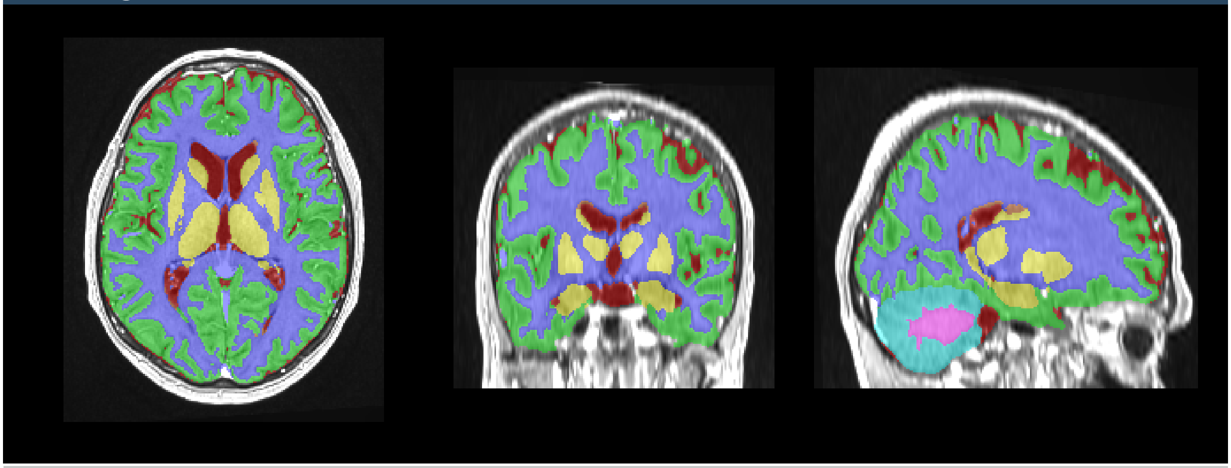

Tissue	Volume (cm^3/%)	
**White Matter (WM)**	469.79 (36.064)	[32.495, 41.493]
Normal Appearing White Matter	467.75 (35.908)	[32.398, 41.404]
Abnormal Appearing White Matter	2.04 (0.157)	[0.000, 0.280]
**Grey Matter (GM)**	708.10 (54.359)	[42.149, 49.393]
Subcortical Grey Matter	39.73 (3.050)	[2.422, 3.082]
Cortical Grey Matter	567.82 (43.590)	[32.543, 38.817]
Cerebellar Grey Matter	100.56 (7.719)	[6.241, 8.437]
**Cerebro Spinal Fluid (CSF)**	104.37 (8.012)	[11.489, 20.140]
**Brain (WM+GM)**	1177.90 (90.423)	[78.478, 87.052]
**Intracranial Cavity (IC)**	1302.65 (100.000)	[100.000, 100.000]

**All the volumes are presented in absolute value (measured in cm^3) and in relative value (measured in relation to the ICV).*

**The Asymmetry Index is calculated as the difference between right and left volumes divided by their mean (in percent).*

**Segmentation images are located in the MNI space (neurological orientation).*

**Values between brackets show expected limits (95%) of normalized volume in function of sex and age for each measure for reference purpose. Values outside the limits are highlighted in red.*

Macrostructures

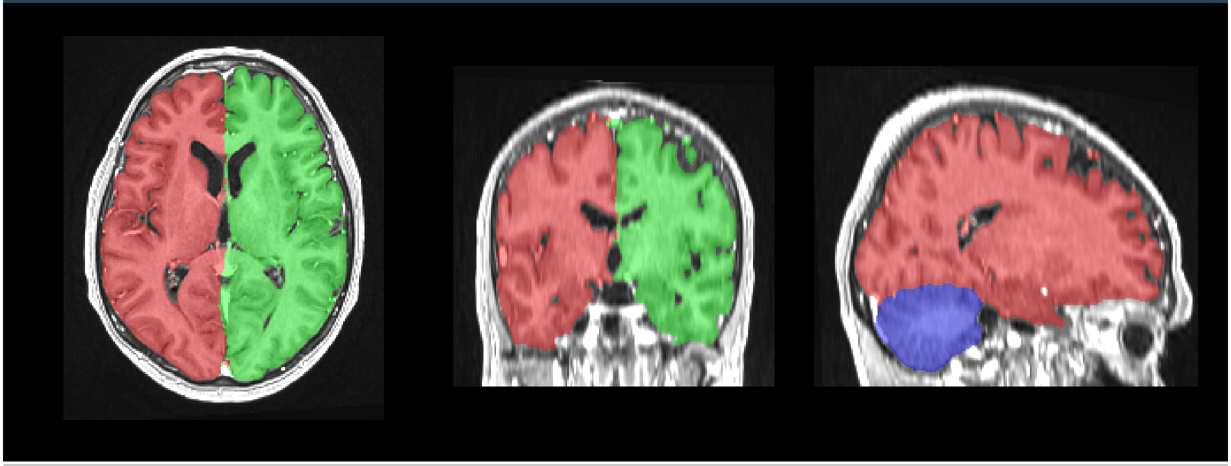

Structure	Total (cm^3/%)	Right (cm^3/%)	Left (cm^3/%)	Asymmetry (%)
**Cerebrum**	1055.34 (81.015)<br>[68.879, 77.455]	531.71 (40.817)<br>[34.465, 38.854]	523.64 (40.198)<br>[34.374, 38.643]	1.5295<br>[-1.164, 1.987]
Cerebrum WM	447.80 (34.376)<br>[30.461, 39.009]	226.44 (17.383)<br>[15.231, 19.617]	221.36 (16.993)<br>[15.213, 19.410]	2.2687<br>[-1.574, 2.877]
Cerebrum GM	607.55 (46.639)<br>[35.168, 41.696]	305.27 (23.434)<br>[17.612, 20.858]	302.28 (23.205)<br>[17.542, 20.851]	0.9846<br>[-1.277, 1.674]
**Cerebellum** *	114.44 (8.786)<br>[7.764, 10.181]	56.89 (4.367)<br>[3.887, 5.126]	57.56 (4.418)<br>[3.867, 5.064]	-1.1668<br>[-2.478, 4.260]
Cerebellum WM	22.00 (1.688)<br>[1.783, 2.733]	10.99 (0.843)<br>[0.903, 1.392]	11.01 (0.845)<br>[0.876, 1.346]	-0.2071<br>[-2.312, 8.698]
Cerebellum GM	100.56 (7.719)<br>[6.241, 8.437]	45.90 (3.524)<br>[2.835, 3.884]	46.55 (3.573)<br>[2.853, 3.857]	-1.3952<br>[-3.882, 4.070]
**Vermis**	8.11 (0.622)<br>[0.501, 0.749]			
**Brainstem**	20.39 (1.565)<br>[1.226, 1.614]			

**Cerebellum volumes does not include vermis volume.*

Structure segmentation

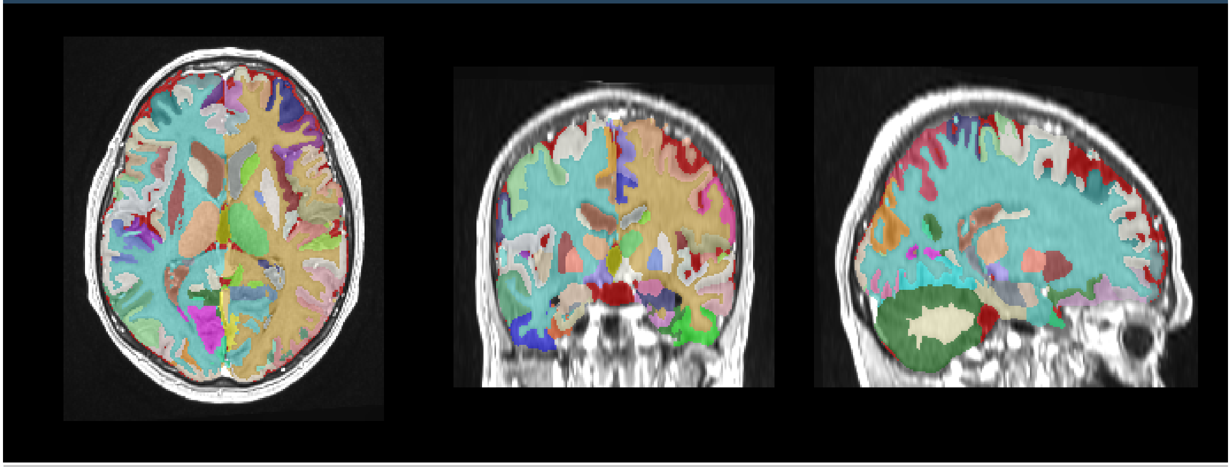

Subcortical	Total (cm^3/%)	Right (cm^3/%)	Left (cm^3/%)	Asymmetry (%)
Accumbens	0.62 (0.047)<br>[0.024, 0.054]	0.33 (0.025)<br>[0.011, 0.026]	0.29 (0.022)<br>[0.013, 0.029]	11.0855<br>[-39.274, 16.423]
Amygdala	2.35 (0.181)<br>[0.108, 0.163]	1.16 (0.089)<br>[0.054, 0.082]	1.19 (0.092)<br>[0.052, 0.083]	-2.8442<br>[-13.670, 14.454]
Basal Forebrain	0.62 (0.047)<br>[0.036, 0.066]	0.32 (0.025)<br>[0.018, 0.034]	0.29 (0.023)<br>[0.016, 0.033]	8.7963<br>[-28.722, 36.947]
Caudate	5.47 (0.420)<br>[0.367, 0.580]	2.66 (0.204)<br>[0.188, 0.294]	2.81 (0.216)<br>[0.177, 0.288]	-5.5657<br>[-4.223, 11.537]
Hippocampus	9.14 (0.702)<br>[0.475, 0.666]	4.51 (0.346)<br>[0.237, 0.340]	4.63 (0.356)<br>[0.234, 0.331]	-2.6787<br>[-8.668, 12.527]
Pallidum	2.64 (0.203)<br>[0.148, 0.229]	1.32 (0.102)<br>[0.074, 0.115]	1.31 (0.101)<br>[0.073, 0.115]	0.7014<br>[-10.043, 12.248]
Putamen	7.23 (0.555)<br>[0.437, 0.660]	3.70 (0.284)<br>[0.215, 0.326]	3.53 (0.271)<br>[0.220, 0.337]	4.5074<br>[-10.438, 5.027]
Thalamus	11.65 (0.895)<br>[0.630, 0.862]	5.80 (0.445)<br>[0.312, 0.427]	5.86 (0.450)<br>[0.315, 0.437]	-1.0383<br>[-8.083, 4.925]
Ventral DC	8.87 (0.681)<br>[0.593, 0.759]	4.43 (0.340)<br>[0.294, 0.380]	4.44 (0.341)<br>[0.297, 0.381]	-0.2409<br>[-5.179, 3.849]

Cortical	Total (cm^3/%)	Right (cm^3/%)	Left (cm^3/%)	Asymmetry (%)
**Frontal lobe**	190.36 (14.613) [10.473, 12.906]	97.51 (7.485) [5.166, 6.426]	92.85 (7.128) [5.277, 6.510]	4.8926 [-6.060, 2.725]
Frontal pole	8.41 (0.645) [0.283, 0.515]	4.09 (0.314) [0.145, 0.262]	4.32 (0.331) [0.129, 0.264]	-5.3166 [-20.490, 26.964]
Gyrus rectus	5.38 (0.413) [0.178, 0.315]	2.83 (0.217) [0.089, 0.170]	2.55 (0.196) [0.079, 0.154]	10.2116 [-18.921, 39.554]
Opercular inf. frontal gyrus	5.97 (0.458) [0.323, 0.559]	3.15 (0.242) [0.153, 0.301]	2.82 (0.217) [0.139, 0.289]	10.8527 [-33.994, 45.286]
Orbital inf. frontal gyrus	2.75 (0.211) [0.098, 0.265]	1.30 (0.100) [0.040, 0.140]	1.45 (0.111) [0.034, 0.150]	-10.2484 [-75.258, 74.264]
Triangular inf. frontal gyrus	6.75 (0.518) [0.327, 0.565]	3.59 (0.276) [0.150, 0.307]	3.15 (0.242) [0.144, 0.291]	13.1026 [-33.496, 42.754]
Medial frontal cortex	2.94 (0.226) [0.147, 0.276]	1.54 (0.118) [0.066, 0.156]	1.40 (0.107) [0.061, 0.141]	9.6298 [-39.244, 57.508]
Middle frontal gyrus	39.70 (3.047) [2.192, 2.947]	19.97 (1.533) [1.082, 1.509]	19.73 (1.515) [1.074, 1.474]	1.1727 [-10.355, 13.502]
Anterior orbital gyrus	4.44 (0.341) [0.181, 0.327]	2.29 (0.176) [0.089, 0.179]	2.15 (0.165) [0.076, 0.164]	6.1499 [-27.314, 49.858]
Lateral orbital gyrus	4.89 (0.375) [0.178, 0.370]	2.40 (0.184) [0.080, 0.192]	2.48 (0.191) [0.080, 0.196]	-3.2347 [-43.366, 40.876]
Medial orbital gyrus	10.62 (0.815) [0.452, 0.654]	5.21 (0.400) [0.217, 0.332]	5.41 (0.415) [0.224, 0.341]	-3.7933 [-24.617, 15.062]
Posterior orbital gyrus	6.06 (0.465) [0.290, 0.500]	2.94 (0.226) [0.128, 0.251]	3.12 (0.240) [0.147, 0.264]	-6.0122 [-37.517, 20.184]
Precentral gyrus	30.12 (2.312) [1.457, 1.983]	14.93 (1.146) [0.706, 1.011]	15.19 (1.166) [0.719, 1.005]	-1.7207 [-15.411, 14.511]
Precentral gyrus medial segment	6.53 (0.502) [0.296, 0.454]	3.51 (0.270) [0.136, 0.233]	3.02 (0.232) [0.141, 0.240]	15.1634 [-32.795, 26.642]
Subcallosal area	3.51 (0.270) [0.113, 0.239]	1.74 (0.133) [0.053, 0.121]	1.77 (0.136) [0.057, 0.121]	-2.1488 [-24.279, 18.075]
Sup. frontal gyrus	30.66 (2.354) [1.565, 2.225]	15.58 (1.196) [0.769, 1.125]	15.08 (1.158) [0.762, 1.134]	3.2826 [-15.107, 14.961]
Sup. frontal gyrus medial segment	10.91 (0.837) [0.668, 1.015]	6.35 (0.488) [0.330, 0.558]	4.56 (0.350) [0.290, 0.506]	32.9418 [-19.816, 41.655]
Supplementary motor cortex	10.72 (0.823) [0.551, 0.841]	6.08 (0.466) [0.269, 0.439]	4.64 (0.356) [0.251, 0.433]	26.7499 [-24.194, 31.183]

**Temporal lobe**	113.07 (8.680) [5.835, 7.442]	55.55 (4.264) [2.884, 3.715]	57.51 (4.415) [2.919, 3.759]	-3.4739 [-7.769, 5.412]
Fusiform gyrus	14.82 (1.138) [0.896, 1.351]	7.17 (0.550) [0.434, 0.695]	7.65 (0.587) [0.433, 0.685]	-6.5322 [-20.792, 20.317]
Planum polare	3.49 (0.268) [0.204, 0.317]	1.70 (0.131) [0.095, 0.162]	1.78 (0.137) [0.097, 0.166]	-4.6113 [-29.684, 25.520]
Planum temporale	3.84 (0.295) [0.191, 0.355]	1.60 (0.123) [0.077, 0.172]	2.25 (0.172) [0.094, 0.202]	-33.8028 [-59.103, 23.811]
Inf. temporal gyrus	26.76 (2.054) [1.320, 1.916]	12.60 (0.967) [0.624, 0.953]	14.16 (1.087) [0.654, 1.004]	-11.6195 [-24.459, 14.772]
Middle temporal gyrus	27.12 (2.082) [1.577, 2.159]	13.47 (1.034) [0.793, 1.128]	13.65 (1.048) [0.738, 1.079]	-1.3331 [-11.500, 22.503]
Sup. temporal gyrus	14.85 (1.140) [0.783, 1.152]	7.43 (0.571) [0.394, 0.614]	7.42 (0.570) [0.350, 0.576]	0.1725 [-17.421, 32.485]
Transverse temporal gyrus	3.01 (0.231) [0.142, 0.285]	1.64 (0.126) [0.060, 0.138]	1.36 (0.105) [0.071, 0.158]	18.8018 [-52.371, 22.521]
Temporal pole	19.18 (1.472) [0.953, 1.511]	9.93 (0.762) [0.488, 0.767]	9.24 (0.710) [0.446, 0.764]	7.1940 [-13.236, 20.738]
**Parietal lobe**	112.15 (8.609) [6.708, 8.385]	56.62 (4.346) [3.324, 4.190]	55.53 (4.263) [3.348, 4.231]	1.9410 [-7.114, 5.372]
Angular gyrus	22.25 (1.708) [0.954, 1.634]	11.16 (0.857) [0.504, 0.880]	11.09 (0.851) [0.419, 0.784]	0.6399 [-7.367, 35.551]
Postcentral gyrus	23.16 (1.778) [1.089, 1.531]	11.75 (0.902) [0.509, 0.774]	11.41 (0.876) [0.543, 0.794]	3.0120 [-23.528, 15.103]
Postcentral gyrus medial segment	1.77 (0.136) [0.079, 0.162]	0.63 (0.048) [0.034, 0.087]	1.14 (0.088) [0.033, 0.087]	-57.9454 [-52.984, 56.554]
Precuneus	24.05 (1.847) [1.254, 1.779]	12.40 (0.952) [0.606, 0.898]	11.65 (0.895) [0.627, 0.902]	6.2321 [-15.297, 11.723]
Sup. parietal lobule	24.31 (1.866) [1.080, 1.563]	12.67 (0.973) [0.516, 0.794]	11.64 (0.893) [0.529, 0.804]	8.5108 [-21.266, 17.562]
Supramarginal gyrus	16.61 (1.275) [0.866, 1.286]	8.00 (0.614) [0.417, 0.659]	8.60 (0.660) [0.411, 0.665]	-7.2442 [-23.261, 23.270]

**Occipital lobe**	75.96 (5.831) [4.307, 5.758]	39.21 (3.010) [2.140, 2.928]	36.76 (2.822) [2.103, 2.894]	6.4526 [-10.672, 13.410]
Calcarine cortex	7.72 (0.592) [0.284, 0.586]	4.22 (0.324) [0.137, 0.297]	3.50 (0.268) [0.136, 0.300]	18.7252 [-27.099, 26.564]
Cuneus	8.07 (0.620) [0.443, 0.726]	3.75 (0.288) [0.212, 0.375]	4.32 (0.331) [0.207, 0.375]	-13.9871 [-28.030, 29.534]
Lingual gyrus	17.78 (1.365) [0.969, 1.378]	9.45 (0.725) [0.480, 0.705]	8.33 (0.639) [0.460, 0.700]	12.6061 [-16.446, 20.760]
Occipital fusiform gyrus	7.55 (0.580) [0.331, 0.599]	4.29 (0.330) [0.159, 0.314]	3.26 (0.250) [0.149, 0.310]	27.4295 [-31.806, 37.811]
Inf. occipital gyrus	12.22 (0.938) [0.678, 1.032]	6.52 (0.500) [0.310, 0.523]	5.70 (0.438) [0.334, 0.543]	13.3520 [-32.204, 21.310]
Middle occipital gyrus	9.84 (0.755) [0.491, 0.860]	4.47 (0.343) [0.224, 0.416]	5.37 (0.412) [0.241, 0.469]	-18.3818 [-37.979, 18.043]
Sup. occipital gyrus	8.66 (0.665) [0.393, 0.665]	4.73 (0.363) [0.202, 0.360]	3.93 (0.302) [0.171, 0.330]	18.3730 [-21.589, 42.030]
Occipital pole	4.13 (0.317) [0.183, 0.449]	1.78 (0.137) [0.075, 0.215]	2.35 (0.180) [0.090, 0.252]	-27.6456 [-60.041, 27.997]
**Limbic cortex**	44.83 (3.442) [2.368, 3.171]	21.19 (1.627) [1.191, 1.627]	23.64 (1.815) [1.129, 1.593]	-10.9425 [-10.327, 17.222]
Entorhinal area	4.59 (0.352) [0.215, 0.339]	2.27 (0.175) [0.104, 0.176]	2.31 (0.178) [0.101, 0.173]	-1.7375 [-22.402, 26.855]
Anterior cingulate gyrus	12.52 (0.961) [0.537, 0.928]	5.65 (0.434) [0.231, 0.479]	6.87 (0.527) [0.268, 0.487]	-19.4803 [-38.527, 26.102]
Middle cingulate gyrus	11.30 (0.867) [0.575, 0.848]	5.30 (0.407) [0.276, 0.436]	6.00 (0.461) [0.271, 0.441]	-12.3976 [-24.282, 24.690]
Posterior cingulate gyrus	9.74 (0.748) [0.529, 0.771]	4.76 (0.365) [0.247, 0.390]	4.98 (0.383) [0.267, 0.395]	-4.6770 [-22.400, 14.290]
Parahippocampal gyrus	6.69 (0.513) [0.316, 0.482]	3.21 (0.246) [0.148, 0.238]	3.48 (0.267) [0.161, 0.254]	-7.9421 [-25.468, 9.570]
**Insular cortex**	31.46 (2.415) [1.721, 2.285]	15.43 (1.184) [0.874, 1.177]	16.03 (1.230) [0.832, 1.124]	-3.8062 [-4.291, 13.835]
Anterior insula	9.08 (0.697) [0.472, 0.698]	4.70 (0.361) [0.232, 0.350]	4.38 (0.336) [0.236, 0.353]	7.1020 [-12.376, 9.752]
Posterior insula	4.41 (0.339) [0.242, 0.376]	2.14 (0.164) [0.121, 0.195]	2.27 (0.174) [0.116, 0.186]	-5.9023 [-12.285, 21.299]
Central operculum	9.33 (0.717) [0.449, 0.639]	4.62 (0.355) [0.210, 0.322]	4.72 (0.362) [0.224, 0.332]	-2.0590 [-23.812, 15.069]
Frontal operculum	3.72 (0.286) [0.195, 0.340]	2.00 (0.153) [0.091, 0.171]	1.73 (0.133) [0.090, 0.183]	14.3731 [-38.860, 27.352]
Parietal operculum	4.90 (0.376) [0.207, 0.389]	1.97 (0.151) [0.081, 0.183]	2.93 (0.225) [0.111, 0.221]	-39.2567 [-59.341, 13.330]

CSF	Total (cm^3/%)	Right (cm^3/%)	Left (cm^3/%)	Asymmetry (%)
Inf. Lateral Ventricle	0.26 (0.020) [0.009, 0.097]	0.16 (0.012) [0.002, 0.056]	0.10 (0.008) [0.003, 0.046]	48.3516 [-86.122, 120.3]
Lateral Ventricle	20.21 (1.551) [0.764, 3.320]	7.83 (0.601) [0.338, 1.676]	12.37 (0.950) [0.371, 1.698]	-44.9345 [-57.302, 49.285]
3rd Ventricle	2.30 (0.177) [0.056, 0.173]			
4th Ventricle	1.63 (0.125) [0.056, 0.209]			
External CSF	79.98 (6.140) [9.332, 17.613]			

Cerebellar vermis	Total (cm^3/%)
Lobules I-V	3.49 (0.268) [0.206, 0.358]
Lobules VI-VII	1.70 (0.131) [0.105, 0.183]
Lobules VIII-X	2.91 (0.223) [0.152, 0.245]

Cortical thickness

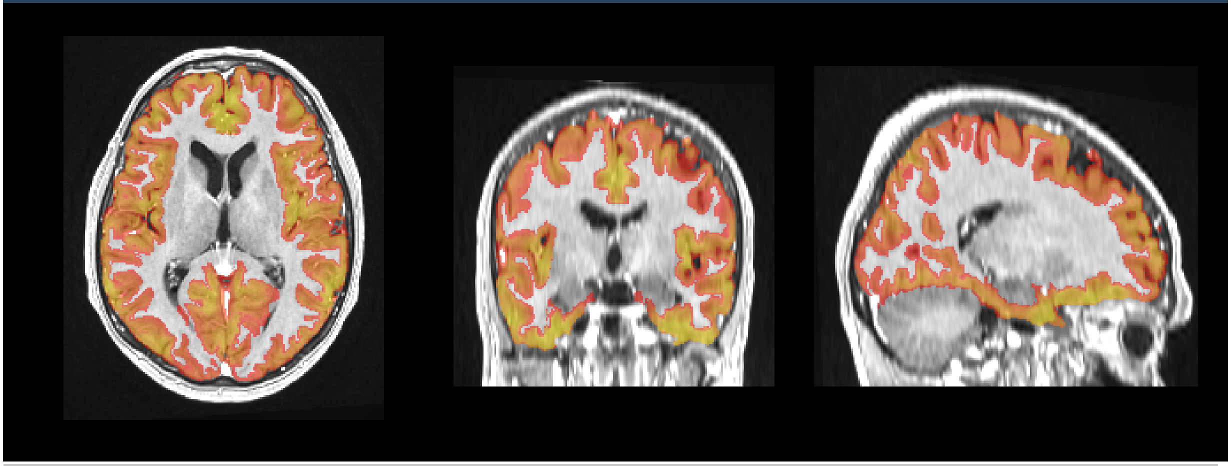

Thickness	Total (mm/norm.)	Right (mm/norm.)	Left (mm/norm.)	Asymmetry (%)
**Frontal lobe**	2.89 (0.026) [0.015, 0.022]	2.88 (0.026) [0.015, 0.022]	2.90 (0.027) [0.015, 0.022]	-0.9209 [-8.355, 4.143]
Frontal pole	3.42 (0.031) [0.015, 0.025]	3.41 (0.031) [0.015, 0.026]	3.43 (0.031) [0.014, 0.025]	-0.7070 [-14.047, 25.761]
Gyrus rectus	4.51 (0.041) [0.016, 0.033]	4.40 (0.040) [0.016, 0.034]	4.63 (0.042) [0.014, 0.033]	-5.0223 [-21.387, 31.825]
Opercular inf. frontal gyrus	2.35 (0.022) [0.012, 0.019]	2.13 (0.019) [0.011, 0.020]	2.60 (0.024) [0.011, 0.019]	-19.9381 [-19.755, 25.995]
Orbital inf. frontal gyrus	2.70 (0.025) [0.014, 0.023]	2.99 (0.027) [0.013, 0.024]	2.44 (0.022) [0.013, 0.023]	20.2039 [-21.179, 27.412]
Triangular inf. frontal gyrus	2.82 (0.026) [0.013, 0.021]	2.75 (0.025) [0.013, 0.022]	2.91 (0.027) [0.012, 0.021]	-5.7888 [-15.412, 27.974]
Medial frontal cortex	4.34 (0.040) [0.020, 0.031]	4.34 (0.040) [0.020, 0.032]	4.34 (0.040) [0.019, 0.031]	0.1770 [-15.428, 26.294]
Middle frontal gyrus	2.71 (0.025) [0.014, 0.024]	2.68 (0.024) [0.014, 0.024]	2.75 (0.025) [0.014, 0.024]	-2.6691 [-11.262, 16.305]
Anterior orbital gyrus	3.16 (0.029) [0.020, 0.032]	3.05 (0.028) [0.019, 0.032]	3.28 (0.030) [0.020, 0.033]	-7.3159 [-24.249, 17.516]
Lateral orbital gyrus	3.12 (0.029) [0.017, 0.028]	3.13 (0.029) [0.017, 0.029]	3.12 (0.029) [0.016, 0.028]	0.3095 [-17.742, 22.348]
Medial orbital gyrus	4.03 (0.037) [0.018, 0.031]	4.02 (0.037) [0.017, 0.031]	4.05 (0.037) [0.018, 0.030]	-0.7627 [-18.663, 17.223]
Posterior orbital gyrus	3.51 (0.032) [0.020, 0.033]	3.91 (0.036) [0.020, 0.033]	3.14 (0.029) [0.019, 0.033]	21.8581 [-14.909, 18.544]
Precentral gyrus	1.96 (0.018) [0.010, 0.016]	1.86 (0.017) [0.010, 0.016]	2.07 (0.019) [0.010, 0.016]	-11.0783 [-13.470, 15.014]
Precentral gyrus medial segment	2.90 (0.027) [0.009, 0.018]	2.74 (0.025) [0.009, 0.018]	3.08 (0.028) [0.009, 0.018]	-11.5968 [-24.509, 19.175]
Subcallosal area	3.92 (0.036) [0.009, 0.028]	3.73 (0.034) [0.007, 0.028]	4.09 (0.037) [0.010, 0.029]	-9.1296 [-43.420, 18.984]

Sup. frontal gyrus	2.39 (0.022) [0.012, 0.020]	2.34 (0.021) [0.012, 0.020]	2.45 (0.022) [0.012, 0.020]	-4.7787 [-9.377, 10.899]
Sup. frontal gyrus medial segment	3.67 (0.034) [0.018, 0.027]	3.86 (0.035) [0.018, 0.028]	3.40 (0.031) [0.017, 0.027]	12.6939 [-10.514, 20.390]
Supplementary motor cortex	3.47 (0.032) [0.014, 0.024]	3.58 (0.033) [0.014, 0.024]	3.34 (0.031) [0.014, 0.024]	6.8512 [-14.767, 17.190]
**Temporal lobe**	3.59 (0.033) [0.011, 0.018]	3.60 (0.033) [0.011, 0.019]	3.58 (0.033) [0.011, 0.018]	0.6868 [-6.153, 11.131]
Fusiform gyrus	4.12 (0.038) [0.025, 0.036]	4.12 (0.038) [0.025, 0.036]	4.12 (0.038) [0.025, 0.037]	0.0558 [-11.634, 9.060]
Planum polare	2.00 (0.018) [0.007, 0.017]	1.76 (0.016) [0.006, 0.017]	2.24 (0.020) [0.006, 0.017]	-24.0005 [-29.784, 22.523]
Planum temporale	2.64 (0.024) [0.010, 0.019]	2.59 (0.024) [0.009, 0.019]	2.67 (0.024) [0.010, 0.020]	-3.3942 [-34.879, 18.665]
Inf. temporal gyrus	3.86 (0.035) [0.023, 0.034]	3.92 (0.036) [0.022, 0.034]	3.81 (0.035) [0.024, 0.035]	2.7133 [-14.909, 6.158]
Middle temporal gyrus	3.56 (0.033) [0.021, 0.030]	3.65 (0.033) [0.020, 0.030]	3.47 (0.032) [0.021, 0.030]	5.0182 [-12.338, 9.124]
Sup. temporal gyrus	3.00 (0.027) [0.016, 0.024]	2.96 (0.027) [0.016, 0.024]	3.03 (0.028) [0.016, 0.024]	-2.4355 [-16.980, 14.965]
Transverse temporal gyrus	2.45 (0.022) [0.008, 0.018]	2.38 (0.022) [0.007, 0.018]	2.53 (0.023) [0.007, 0.019]	-5.9952 [-30.573, 19.328]
Temporal pole	3.96 (0.036) [0.024, 0.035]	3.93 (0.036) [0.024, 0.036]	4.00 (0.037) [0.023, 0.036]	-1.7931 [-12.901, 12.823]
**Parietal lobe**	2.36 (0.022) [0.021, 0.030]	2.29 (0.021) [0.022, 0.030]	2.43 (0.022) [0.021, 0.030]	-5.6191 [-4.677, 8.194]
Angular gyrus	2.65 (0.024) [0.013, 0.022]	2.57 (0.023) [0.013, 0.022]	2.74 (0.025) [0.013, 0.022]	-6.4854 [-16.152, 20.680]
Postcentral gyrus	1.42 (0.013) [0.006, 0.012]	1.34 (0.012) [0.006, 0.012]	1.50 (0.014) [0.006, 0.012]	-11.5049 [-23.441, 15.982]
Postcentral gyrus medial segment	1.79 (0.016) [0.004, 0.012]	2.40 (0.022) [0.003, 0.013]	1.46 (0.013) [0.003, 0.012]	48.8736 [-43.560, 52.258]
Precuneus	3.31 (0.030) [0.015, 0.026]	3.30 (0.030) [0.014, 0.025]	3.32 (0.030) [0.015, 0.026]	-0.5554 [-18.291, 7.699]
Sup. parietal lobule	1.96 (0.018) [0.007, 0.014]	1.88 (0.017) [0.006, 0.014]	2.04 (0.019) [0.007, 0.015]	-8.4856 [-26.851, 10.347]
Supramarginal gyrus	2.56 (0.023) [0.013, 0.021]	2.42 (0.022) [0.012, 0.020]	2.70 (0.025) [0.012, 0.021]	-10.9440 [-21.298, 14.246]

**Occipital lobe**	2.52 (0.023) [0.012, 0.022]	2.46 (0.023) [0.012, 0.022]	2.58 (0.024) [0.012, 0.022]	-4.8618 [-7.146, 10.356]
Calcarine cortex	2.61 (0.024) [0.004, 0.018]	2.59 (0.024) [0.004, 0.018]	2.63 (0.024) [0.005, 0.018]	-1.6474 [-28.906, 21.748]
Cuneus	2.52 (0.023) [0.007, 0.017]	2.38 (0.022) [0.006, 0.017]	2.64 (0.024) [0.006, 0.018]	-10.2439 [-25.542, 19.670]
Lingual gyrus	2.69 (0.025) [0.014, 0.026]	2.66 (0.024) [0.014, 0.026]	2.72 (0.025) [0.014, 0.027]	-2.2587 [-15.686, 10.405]
Occipital fusiform gyrus	2.12 (0.019) [0.014, 0.025]	2.22 (0.020) [0.014, 0.026]	1.98 (0.018) [0.013, 0.025]	11.2654 [-14.181, 26.345]
Inf. occipital gyrus	2.58 (0.024) [0.015, 0.024]	2.49 (0.023) [0.014, 0.024]	2.68 (0.025) [0.015, 0.025]	-7.2905 [-20.361, 11.287]
Middle occipital gyrus	3.24 (0.030) [0.015, 0.026]	3.19 (0.029) [0.014, 0.026]	3.28 (0.030) [0.014, 0.027]	-2.7461 [-20.645, 17.574]
Sup. occipital gyrus	2.01 (0.018) [0.007, 0.016]	1.84 (0.017) [0.007, 0.017]	2.22 (0.020) [0.006, 0.016]	-18.7761 [-20.499, 31.491]
Occipital pole	1.56 (0.014) [0.004, 0.016]	1.57 (0.014) [0.004, 0.016]	1.55 (0.014) [0.004, 0.016]	1.2582 [-35.171, 29.956]
**Limbic cortex**	4.09 (0.037) [0.021, 0.030]	4.06 (0.037) [0.021, 0.030]	4.12 (0.038) [0.021, 0.030]	-1.3122 [-7.418, 6.028]
Entorhinal area	3.79 (0.035) [0.024, 0.032]	3.66 (0.034) [0.024, 0.033]	3.91 (0.036) [0.023, 0.032]	-6.6826 [-15.222, 18.715]
Anterior cingulate gyrus	4.54 (0.042) [0.024, 0.035]	4.49 (0.041) [0.024, 0.035]	4.57 (0.042) [0.023, 0.035]	-1.6641 [-10.321, 12.671]
Middle cingulate gyrus	4.21 (0.039) [0.017, 0.028]	4.20 (0.038) [0.017, 0.028]	4.22 (0.039) [0.016, 0.029]	-0.4317 [-11.875, 15.925]
Posterior cingulate gyrus	3.89 (0.036) [0.019, 0.030]	3.87 (0.035) [0.019, 0.030]	3.91 (0.036) [0.019, 0.030]	-1.0429 [-13.315, 8.275]
Parahippocampal gyrus	3.56 (0.033) [0.019, 0.028]	3.65 (0.033) [0.019, 0.029]	3.48 (0.032) [0.018, 0.028]	4.8176 [-12.592, 17.657]
**Insular cortex**	3.43 (0.031) [0.018, 0.027]	3.52 (0.032) [0.017, 0.027]	3.33 (0.031) [0.018, 0.027]	5.6171 [-10.637, 6.128]
Anterior insula	4.05 (0.037) [0.024, 0.033]	4.21 (0.039) [0.024, 0.034]	3.88 (0.036) [0.023, 0.033]	8.2537 [-8.170, 11.319]
Posterior insula	3.71 (0.034) [0.015, 0.027]	3.78 (0.035) [0.015, 0.028]	3.65 (0.033) [0.015, 0.027]	3.6336 [-12.941, 20.452]
Central operculum	2.97 (0.027) [0.014, 0.024]	3.12 (0.029) [0.014, 0.025]	2.83 (0.026) [0.014, 0.024]	9.7847 [-14.356, 16.156]
Frontal operculum	3.53 (0.032) [0.016, 0.026]	3.55 (0.033) [0.015, 0.027]	3.50 (0.032) [0.015, 0.026]	1.6010 [-15.900, 17.193]
Parietal operculum	2.80 (0.026) [0.012, 0.022]	2.53 (0.023) [0.012, 0.022]	2.99 (0.027) [0.012, 0.023]	-16.5941 [-25.737, 21.514]
