# Supplementary material for: Magnetic Resonance Imaging-Based Cortical and Subcortical Volumetric Changes in Alzheimer’s Disease: Association with Clinical Severity
Source: Diagnostics (Basel). 2026 Aug 16;16(16):2596. doi: 10.3390/diagnostics16162596 (PMC13511431; doi:10.3390/diagnostics16162596)
Supplement: Supplementary file 1 [file diagnostics-16-02596-s001.zip › Supplementary Material S2.pdf]

**Supplementary Material S2:** Internal Validation and DeLong Comparisons of the ROC Models Presented in Tables 6 and 7

| Comparison | Model | Apparent AUC | Optimism-corrected AUC | 5-fold CV AUC (mean ± SD) | Events per variable | ΔAUC vs. CSF | DeLong p |
|---|---|---|---|---|---|---|---|
| **AD vs. HC (Table 6, Figure 3)** | Hippocampus + CSF | 0.847 | 0.837 | 0.826 ± 0.008 | 17.0 | +0.010 | 0.418 |
| **AD vs. HC (Table 6, Figure 3)** | Hippocampus + ACgG | 0.742 | 0.724 | 0.709 ± 0.013 | 17.0 | -0.095 | 0.130 |
| **AD vs. HC (Table 6, Figure 3)** | Hippocampus + ACgG + CSF | 0.834 | 0.812 | 0.799 ± 0.013 | 11.3 | -0.003 | 0.878 |
| **AD vs. HC (Table 6, Figure 3)** | Hippocampus + ACgG + CSF + PHG cortical thickness | 0.831 | 0.803 | 0.784 ± 0.016 | 8.5 | -0.005 | 0.785 |
| **MCI vs. HC (Table 7, Figure 5)** | Hippocampus + CSF | 0.811 | 0.797 | 0.776 ± 0.011 | 17.0 | +0.127 | 0.033 |
| **MCI vs. HC (Table 7, Figure 5)** | Hippocampus + ACgG | 0.710 | 0.686 | 0.663 ± 0.022 | 17.0 | +0.026 | 0.781 |
| **MCI vs. HC (Table 7, Figure 5)** | Hippocampus + ACgG + CSF | 0.826 | 0.801 | 0.776 ± 0.019 | 11.3 | +0.142 | 0.026 |
| **MCI vs. HC (Table 7, Figure 5)** | Hippocampus + ACgG + CSF + PHG cortical thickness | 0.822 | 0.788 | 0.769 ± 0.021 | 8.5 | +0.138 | 0.031 |

Optimism correction was based on 1000 bootstrap replications; cross-validation comprised 20 repetitions of 5-fold partitioning. ΔAUC compares each combined model with CSF volume, the strongest single marker in both analyses. Cited in Results in the footnotes of Tables 6 and 7.
