# Supplementary material for: Magnetic Resonance Imaging-Based Cortical and Subcortical Volumetric Changes in Alzheimer’s Disease: Association with Clinical Severity
Source: Diagnostics (Basel). 2026 Aug 16;16(16):2596. doi: 10.3390/diagnostics16162596 (PMC13511431; doi:10.3390/diagnostics16162596)
Supplement: Supplementary file 1 [file diagnostics-16-02596-s001.zip › Supplementary Material S3.pdf]

Supplementary Material S3: Pooled, Group-Adjusted, and Within-Group Correlations Between MMSE Score and Volumetric Measures

Measure	Pooled ρ	p	Group-adjusted partial ρ	p	AD (n=34)	MCI (n=34)	HC (n=34)
**Hippocampus**	+0.32	0.001	+0.23	0.023	+0.40 (0.019)	+0.30 (0.081)	-0.04 (0.824)
**ACgG**	+0.34	<0.001	+0.11	0.275	+0.45 (0.008)	+0.06 (0.737)	-0.04 (0.822)
**Thalamus**	+0.24	0.013	+0.03	0.747	+0.28 (0.112)	+0.11 (0.524)	-0.36 (0.038)
**Precuneus**	+0.19	0.059	+0.17	0.100	+0.43 (0.010)	+0.14 (0.414)	-0.11 (0.538)
**Total CSF**	-0.54	<0.001	-0.31	0.002	-0.42 (0.013)	-0.45 (0.007)	-0.07 (0.696)
**Lateral ventricle**	-0.40	<0.001	-0.23	0.019	-0.39 (0.024)	-0.27 (0.129)	-0.07 (0.694)

Spearman rank correlation; partial correlations were computed on rank-transformed data controlling for groups. Volumes are expressed as %TIV. Within-group cells show ρ (p). Cited in Results in Figure 7.
